# Genomics combined with UAS data enhances prediction of grain yield in winter wheat

**DOI:** 10.3389/fgene.2023.1124218

**Published:** 2023-03-29

**Authors:** Osval A. Montesinos-López, Andrew W. Herr, José Crossa, Arron H. Carter

**Affiliations:** ^1^ Facultad de Telemática, Universidad de Colima, Colima, México; ^2^ Department of Crop and Soil Sciences, Washington State University, Pullman, WA, United States; ^3^ International Maize and Wheat Improvement Center (CIMMYT), Texcoco, Edo. de México, México; ^4^ Colegio de Postgraduados, Montecillos, Edo. de México, México

**Keywords:** high throughput phenotyping, genomic prediction, winter wheat, selection accuracy, genomic selection

## Abstract

With the human population continuing to increase worldwide, there is pressure to employ novel technologies to increase genetic gain in plant breeding programs that contribute to nutrition and food security. Genomic selection (GS) has the potential to increase genetic gain because it can accelerate the breeding cycle, increase the accuracy of estimated breeding values, and improve selection accuracy. However, with recent advances in high throughput phenotyping in plant breeding programs, the opportunity to integrate genomic and phenotypic data to increase prediction accuracy is present. In this paper, we applied GS to winter wheat data integrating two types of inputs: genomic and phenotypic. We observed the best accuracy of grain yield when combining both genomic and phenotypic inputs, while only using genomic information fared poorly. In general, the predictions with only phenotypic information were very competitive to using both sources of information, and in many cases using only phenotypic information provided the best accuracy. Our results are encouraging because it is clear we can enhance the prediction accuracy of GS by integrating high quality phenotypic inputs in the models.

## Introduction

Agriculture needs to provide a significant increase in food, fuel, fiber, and fine chemicals in the next century to meet the needs of the growing world population. Different challenges exist in meeting these needs because of the effects of climate change, including an increased risk of drought and high temperatures, torrential rains, degradation of arable land, and the depletion of water resources (Atefi, et al., 2021). To mitigate these challenges, plant breeders are working to develop high-yielding, stress-tolerant crop varieties adapted to future climatic conditions and resistant to new pests and diseases (Fischer, 2009; Furbank and Tester, 2011; Rahaman et al., 2015).

Marker technology has been used in plant breeding since the 1980s, but it was not until 2001 when genomic selection (GS) was proposed by Meuwissen et al. (2001) to estimate all marker effects. Using this strategy, the full potential of markers was engaged, since GS is able to predict the output variable using all markers simultaneously in the model. The applications for GS continue growing, as it is employed by breeders in wheat (*Triticum aestivum* L.), maize (*Zea mays* L.), cassava (*Manihot esculenta* L.), rice (*Oryza sativa* L.), chickpea (*Cicer arietinum* L.), groundnut (*Arachis hypogaea* L.), etc. (Roorkiwal et al., 2016; Crossa et al., 2017; Wolfe et al., 2017; Huang et al., 2019). However, implementing GS in many plant breeding programs is challenging due to the many factors that affect its accuracy. Some of these factors include 1) genotyping quality, 2) not following the guidelines about where in the breeding program GS can be efficiently applied (Crossa et al., 2017; Yoosefzadeh-Najafabadi, et al., 2022), 3) insufficient number of lines in the reference (training) population, 4) appropriate allocation of samples and SNPs to training, testing, and validation using difference methods such as cross validation (Montesinos-López et al., 2022), 5) organization of field designs, 6) cross-validation strategy, tested lines in tested environments, tested lines in untested environments, etc., 7) heritability of the trait, 8) population structure, and 9) prediction model, etc.

There is empirical evidence that integrating high throughput phenotyping information collected by unmanned aerial systems (UASs), handheld scanners, tractor-mounted systems, and low orbiting satellite systems, in combination with genomic data, has the potential to complement GS and increase crop productivity. One of the advantages of recent phenotyping technology is that it can quickly and accurately obtain data on many agronomic traits (Atkinson et al., 2018). While the availability of new classes of phenomic information has fueled the development of a phenomic-based analog to GS, phenomic selection (PS), it is more probable that the integration of high throughput phenotypic information with other omics data is what can significantly improve the accuracy of GS. For example, Wu et al. (2022), using three omics datasets (transcriptomics, genomics, and metabolomics) as predictors, found that the integration of the three sources of information improves prediction accuracy in barley (*Hordeum vulgare* L.). Also, Hu et al. (2021) found that integrating multi-omics (transcriptomic, metabolomic, and genomics) data improved prediction accuracies of oat (*Avena sativa* L.) agronomic and seed nutritional traits in multi-environment trials and distantly related populations in addition to single-environment predictions.

Because phenotypic variation observed across diverse environments is a product of genetic and environmental variation, environmental information acts as a central bottleneck for the application of modern genomics-assisted prediction tools, especially for use across multiple environments. Thus, it is of paramount importance to incorporate high throughput environmental data into genomic prediction models to improve predictions in new environments with the same environmental characteristics (Rogers et al., 2021). Also, all environmental (historical and non-historical) data should be included as predictors to model the genotype by environment interaction more efficiently, which is key to increasing the prediction performance and genetic gain in breeding programs. The addition of environmental data in the modeling process is fundamental for more accurately predicting cultivars across diverse growing conditions (e.g., Jarquín et al., 2014; Messina et al., 2018; Millet et al., 2019).

As referenced, there continues to be a growing amount of empirical evidence that combining genomic, phenotypic, and environmental data is key to improving prediction accuracy (Montesinos-López et al., 2017; Cuevas et al., 2019; Krause et al., 2019). It is important to note that many robotics systems have been employed to measure plant orientation, plant height, leaf length, leaf area, leaf angle, leaf and stem width, and stalk count of many species such as sorghum (*Sorghum bicolor* L.), maize, cauliflower (*Brassica oleracea* L.), sunflower (*Helianthus annuus* L.), brussels sprouts (*B. oleracea* L.), and savoy cabbage (*B. oleracea* L.) (Jay et al., 2015; Fernandez et al., 2017; Baweja et al., 2018; Vázquez-Arellano et al., 2018; Vijayarangan et al., 2018; Bao et al., 2019; Breitzman et al., 2019; Qiu et al., 2019; Young et al., 2019; Zhang et al., 2020), architectural traits and density of the peanut canopy (Yuan et al., 2018), the number of cotton (*Gossypium sp.*) bolls (Xu et al., 2018), berry size and color of grapes (*Vitis sp.*) (Kicherer et al., 2015), and volume, shape, and yield estimation of vineyards (Lopes et al., 2016; Vidoni et al., 2017). Even the promising results of high throughput phenotyping face many technical challenges that need to be addressed regarding sensing, path planning, localization, obstacle avoidance, and object detection. More research is required to overcome these limitations of phenotyping robots and improve their accuracy, speed, and safety (Atefi, et al., 2021). Some publications that combine genomics and environmental information are Basnet et al. (2019), Monteverde et al. (2019), Washburn et al. (2021), Jarquin et al. (2021), Rogers and Holland (2022), Costa-Neto, et al. (2021a), Costa-Neto, et al. (2021b), among others. Few publications are available that integrate genomics, phenomics, and environmental information (Crossa et al., 2021).

In this study, using data from soft white winter wheat collected from 2019 to 2022 by Washington State University, we evaluated the prediction performance of integrating genomics and high throughput phenotypic information to predict grain yield under two scenarios of cross validation (CV), prediction of partially tested lines in tested environments using 7-fold cross validation (7FCV) and prediction of partially untested lines in untested environments using leave one environment out (LOEO) cross validation. These two strategies of CV were implemented using the Bayesian genomic best linear unbiased predictor (GBLUP) and the partial least squares (PLS) method.

## Materials and methods

### Datasets 1 to 4 (wheat data)

Wheat lines used in this study are from the breeding program of Washington State University (WSU) and were grown at various locations in the state of Washington on grower-cooperator fields using common agricultural practices for the region. Grain yield (GY) was collected using a Zürn 150 Combine (Zürn Harvesting GmbH & Co.) and was used for each of the four data sets.

• Dataset 1, Wheat_1 (Year 2019) contains 1,397 unique lines and three environments (Kincaid, Lind, Pullman) and contains 1,869 total observations since some lines are repeated in various environments.
• Dataset 2, Wheat_2 (Year 2020) contains 758 unique lines and six environments (Farmington, Harrington, Kincaid, Lind, Ritzville, and Walla Walla) and contains 952 total observations since some lines are repeated in various environments.
• Dataset 3, Wheat_3 (Year 2021) contains 452 unique lines and eight environments (Davenport, Harrington, Kahlotus, Kincaid, Lind, Pullman, Ritzville and Walla Walla) and contains 780 total observations since some lines are repeated in various environments.
• Dataset 4, Wheat_4 (Year 2022) contains 363 unique lines and six environments (Davenport, Farmington, Harrington, Prescott, Pullman and Ritzville) and contains 483 total observations since some lines are repeated in various environments.

Phenotypic data was collected using the Sentera Quad Multispectral Sensor (Sentera, St Paul, MN), which covered target bands of interest for winter wheat evaluation. The camera has four sensors that cover eight broad spectral bands between 450 and 970 nm. An unmanned aircraft system (UAS) mounted with the Sentera camera flew a programmed route at an elevation of 45 m capturing overlapping georeferenced images. Collected UAS images were stitched and prepped for data extraction in Pix4Dmapper (Pix4D Inc., Denver, CO), creating a single orthomosaic image for each sensor per location. Orthomosaic images were transferred to Geographic Information System (QGIS) for plot identification and then further processed with a custom R code for calibration, index calculation, and single plot mean data extraction. In 2019, a single reflectance panel (85% reflectance) was used for radiometric calibration on red, blue, green, (RBG) and red edge bands (RE1 and RE2). Quantum efficiency coefficients were used to calculate near infrared (NIR) using:

$$NIR=\left(2.921\times Blue\right)-\left(0.754\times Red\right)$$

The NIR band was then normalized with a coefficient of 3.07 during the calculation of SRIs. In 2020 through 2022, a set of calibration panels were used (five panels ranging from 2%–85% reflectance, MosaicMill Oy, Vantaa, Finland). All raw band layers were adjusted based on the relationship:

SR=DN×Slope±intercept

Where the slope and intercept are based on the regression of the observed reflectance in calibration panels, digital numbers (DN) are the raw observed pixel values, and surface reflectance (SR) is the true reflectance value (Iqbal et al., 2018). All datasets used adjusted multispectral band values to calculate indices for further model analysis.

All the lines were genotyped using genotyping-by-sequencing (GBS; Poland et al., 2012). The original SNPs totaled 6,075,743, but after filtering for SNPs with homozygosity >80%, for less than 50% missing data, greater than a 0.05 minor allele frequency, and less than 5% heterozygosity, we end up with 19,645 SNPs. Markers with missing data were imputed using the “expectation-maximization” algorithm in the “R” package rrBLUP (Endelman, 2011). In each data set, the best linear unbiased estimates (BLUEs) were computed under two experimental designs:

### For trials under an alpha lattice design

The BLUEs for GY within each environment were calculated using the lmer function of the lme4 package (Bates et al., 2015) of the R statistical software with the following mixed linear model:

$$y_{ijkl}=\mu +g_{i}+check_{i}+t_{j}+r_{k\left(j\right)}+b_{l\left(jk\right)}+\varepsilon_{ijkl}$$

where $y_{ijkl}$ is the GY of the *ith* genotype in the *jth* trial, *kth* replicate and *lth* block, μ is the general mean, $g_{i}$ is the fixed effect of the genotype *i*, $check_{i}$ is the fixed effect of the check-genotype *i,* $t_{j}$ is the random effect of the trial, $t_{j}∼NIID\left(0,\sigma_{t}^{2}\right)$ ; where NIID stands for normal, independent and identically distributed, $r_{k\left(j\right)}$ is the random effect of the replicate within the trial, $r_{k\left(j\right)}∼NIID\left(0,\sigma_{r}^{2}\right)$ ; $b_{l\left(jk\right)}$ is the random effect of the incomplete block within the trial and the replicate, $b_{l\left(jk\right)}∼NIID\left(0,\sigma_{b}^{2}\right)$ ; and $\varepsilon_{ijkl}$ is the residual $\varepsilon_{ijkl}∼NIID\left(0,\sigma^{2}\right)$ .

### For trials under an augmented randomized complete block design

In this experimental design, the BLUEs for GY within each environment were calculated using the lmer function of the lme4 package of the R statistical software with the following mixed linear model:

$$y_{ij}=\mu +g_{i}+check_{i}+b_{j}+\varepsilon_{ij}$$

where $y_{ij}$ is the GY of the *ith* genotype in the *jth* block, μ is the general mean, $g_{i}$ is the fixed effect of the genotype *i*, $check_{i}$ is the fixed effect of the check-genotype *i,* $b_{j}$ is the random effect of the *jth* block, $b_{j}∼NIID\left(0,\sigma_{b}^{2}\right)$ ; and $\varepsilon_{ij}$ is the residual $\varepsilon_{ij}∼NIID\left(0,\sigma^{2}\right)$ .

### Bayesian genomic best linear unbiased predictor model

The Bayesian models implemented only differ in the predictor they used. For this reason, the general model is given:

$$Y_{ij}=\mu +{ETA}_{ijk}+\epsilon_{ij}$$ (1)

Where $Y_{ij}$ denotes the response variable in the j*th* line in the i*th* environment, μ denotes the general mean (intercept), and $\epsilon_{ij}$ are random error components assumed to be independent normal random variables with mean 0 and variance $\sigma^{2}.\;{ETA}_{ijk}$ is the predictor for the j*th* line in the i*th* environment. The different ${ETA}_{ijk}´s$ (*ETA*) used are provided in Supplementary Table SA1.

The vector of length eleven $\left[H_{ij1,}\ldots ,H_{ij11}\right]$ of the information include both the raw multispectral data and calculated indices denoted as: Blue, Green, Red, NIR, RE1, RE2, 900, 975 nm, NDRE1, NDVI, and Canopy Cover. While when the vector of length three ( $I_{ij1,}I_{ij2},I_{ij3})$ was used as independent variables including only the indices: Can_Cover, NDRE1, and NDVI were used. The implementation of these models was carried out in the R statistical software (R Core Team, 2022) using the BGLR library of Pérez and de los Campos (2014). Equations used in the calculation of indices can be found in Table 1.

**TABLE 1:** Spectral reflectance indices implemented. The first column provides the name of the spectral indices, the second one its abbreviation, the third shows the equation used for computing each index, and the last one indicates the reference for each index.

Spectral reflectance indices	Abbreviation	Equation	Reference
Normalized Difference	NDVI	$\frac{R800-R680}{R800+R680}$	Rouse et al. (1973)
Vegetation Index			
Normalized Difference	NDRE1	$\frac{R800-R700}{R800+R700}$	Gitelson and Merzlyak (1996)
Red Edge 1			
Percent Canopy Coverage	Canopy Cover	$\frac{1}{N}\sum_{i=1}^{N}{GNDVI}_{i}$	Sankaran et al. (2015)

### Partial least squares model

This study utilized the univariate PLS model, a statistical machine learning method introduced by Wold (2001) in econometrics and chemometrics for regression analysis. PLS is very useful for prediction problems where the number of independent variables ( p) is larger than the number of observations ( n) and when predictors are highly correlated. Under the univariate PLS framework, the response variable $\left(Y\right)$ is a vector instead of a matrix of order n×1 that is linked to a set of explanatory variables ( X ) of order n×p (Wold, 2001; Boulesteix and Strimmer 2006). In PLS, instead of regressing Y on X **,** we regressed Y on T , where T are the latent variables (LVs), also called X **-**scores or latent vectors; these LVs are related to the original X and Y matrices. The goal of PLS regression is to maximize the covariance between Y and T ; however, an iterative procedure is implemented for its computation. The main steps to compute the LVs under a univariate framework using the kernel algorithm for PLS are:

Step 1 Initialization of E = X and F = Y . Center each column of E and F ; scaling is optional.

Step 2 Compute $S=X^{T}Y$ (Cross product matrix) and then $SS^{T}=X^{T}YY^{T}X$ and $S^{T}S=Y^{T}XX^{T}Y$ **.**

Step 3 Compute the singular value decomposition (SVD) of $SS^{T}$ and $S^{T}S$ .

Step 4 Obtain w and q **,** the eigenvectors to the largest eigenvalue of $SS^{T}$ and $S^{T}S$ **,** respectively**.**

Step 5 Compute scores t and u as t=Xw=Ew and u=Yq=Fq .

Step 6 Normalize the t and u scores as $t=t/\sqrt{t^{T}t}$ and $u=u/\sqrt{u^{T}u}$ .

Step 7 Next, compute X and Y loadings as $p=E^{T}t$ and $q=F^{T}t$ **.**

Step 8 Deflate matrices E and F as $E_{n+1}=E_{n}-tp^{T}$ and $F_{n+1}=F_{n}-tq^{T}.$

Step 9 Use as input $E_{n+1}$ and $F_{n+1}$ , of Step 8, in Step 2, and repeat steps 2 to 9 until the deflated matrices are empty or the necessary number of components have been extracted.

With the outputs of w , t , p and q vectors, the matrices **W**, **T**, **P**, and **Q**, respectively, are built. Finally, after having all the columns of W **,** we compute R as:

$$R=W{\left(P^{T}W\right)}^{-1}$$

Next, with R we can compute the LVs, which are related to the original X matrix as:

T=XR

Next, since we regressed Y on T , the resulting beta coefficients are $b={\left(T^{T}T\right)}^{-1}T^{T}Y$ . However, to convert these back to the realm of the original variables ( X) , we pre-multiplied with matrix R the beta coefficients ( b ); since T=XR,

B=R b

To reach optimal performance of the PLS method, only the first a components are used. Since regression and dimension reduction are performed simultaneously, all B , T , W , P and Q are part of the output. Both X and Y are considered when calculating the LVs in T . Thereafter, predictions for new data ( $X_{new}$ ) should be done with:

$${\hat{Y}}_{new}=X_{new}B=X_{new}Rb=T_{new}b$$

where ${T_{new}=X}_{new}R$ . In this study, the optimal number of components was determined by cross-validation. We used the NRMSE, with an inner 10-fold cross-validation for selecting the optimal number of hyperparameters.

In this application, we used the concatenation of the different sources of information for each predictor (ETA1 to ETA9 given in Table 1) as the matrix of independent variables **X.** For this reason, we first computed the design matrices of environments ( $X_{E}),$ the design matrix of genotypes ( $X_{g})$ and the design matrix of the Genotype × Environments term ( $X_{gE}$ ). But, since the PLS method does not allow direct inclusion, like the Bayesian GBLUP model, the genomic relationship matrix of lines $\left(G=\frac{MM^{T}}{r}\right)$ , where M denotes the matrix of markers (coded as 0, 1 and 2) of order J×r ; J denotes the number of lines; r the total number of markers. The design matrices of lines and genotype × environments were post-multiplied by their corresponding square root matrices of their corresponding relationship matrices to incorporate into the design matrix this relationship information. That is, instead of using only $X_{g}$ ; $X_{gE}$ as input, we used $X_{g}L_{g}$ **(**with $L_{g}=G^{0.5})\;and\;X_{gE}L_{gE}$ (with $L_{gE}=G_{GE}^{0.5})$ . For this reason, the final input matrix used for ETA1 to ETA9 under the PLS model was; $X=\left[X_{E},X_{g}L_{g}\right];X=\left[X_{E},X_{g}L_{g},H\right];X=\left[X_{E},X_{g}L_{g},H,X_{gE}L_{gE}\right]$ ; $X=\left[X_{E},X_{g}L_{g},H,X_{gE}L_{gE},X_{g}L_{g}:H\right]$ **;** $X=\left[X_{E},X_{g}L_{g},I\right];X=\left[X_{E},X_{g}L_{g},I,X_{gE}L_{gE}\right]$ ; $X=\left[X_{E},X_{g}L_{g},I,X_{gE}L_{gE},X_{g}L_{g}:I\right]$ ; $X=\left[X_{E},H\right]$ ; $X=\left[X_{E},I\right]$ respectively, where $H={\left.{\left[H\right.}_{11},\ldots ,H_{1J},\ldots ,H_{IJ}\right]}^{T}$ ; $I={\left.{\left[I\right.}_{11},\ldots ,I_{1J},\ldots ,I_{IJ}\right]}^{T}$ ; $X_{g}L_{g}:H$ represents the interaction term between the genomic information and the multispectral information; $X_{g}L_{g}:I$ represents the interaction term between the genomic information and three indices built from the multispectral information. We did not post-multiply the design matrix of environments ( $X_{E})$ since we did not compute an environmental relationship matrix with environmental covariates, only with the dummy values of the position of environments. For this reason, under the PLS model were used as input the vector of response variables ( Y) and the input matrix X **,** just defined above**.** The implementation of the PLS models was performed with the R statistical software (R Core Team 2022) using the PLS library (Mevik and Wehrens, 2007).

### Metrics for evaluation of prediction accuracy

In each of the four datasets (corresponding to years 2019–2022), for implementing the type of cross-validation partially tested lines in tested environments, we used seven-fold cross validation (7FCV) (Montesinos-López et al., 2022). For this reason, 7−1 folds (85.71% of the data) were assigned to the outer-training set and the remaining fold (14.29% of the data) was assigned to the outer-testing set, until each of the 7 folds were tested once. Under the PLS model for tuning the number of principal components required ten nested cross-validations, that is, the outer-training was divided into ten groups where nine were used for inner training set (90% of the training) and one for the validation (inner-testing) set (10% of the outer training). This means that under the PLS model, the data set was divided in outer-testing (14.29% of data), inner-training (77.14% of data), and validation (8.57% of data). Using the validation set, the optimal number of principal components was selected. Also, it is important to point out that the sum of the inner-training plus the validation equals the outer-training. Next, the average of the ten validation folds was reported as the metric of prediction accuracy to select the optimal hyperparameter (number of principal components). Then, using this optimal hyperparameter, the PLS model was refitted with the whole outer-training set (the 7−1 folds), and finally, the prediction of each outer-testing set was obtained. For the selection of the hyperparameters under the inner-training, the average mean square error was computed and used as the metric of accuracy, but for the outer-training to evaluate the prediction accuracy under the partially tested lines in the test environments cross-validation, the average Pearson´s correlation was computed. It is important to note that under the GBLUP, not tuning was required and only the outer 7FCV was implemented and the average of the 7 folds was reported as prediction accuracy for each environment using the Pearson´s correlation. But the computation of Pearson´s correlation across environments (Global) under the outer 7FCV was done between averages of true and predicted phenotypes of lines over environments per year (data set). On the other hand, to implement the cross-validation partially tested lines in untested environments we used a leave one environment out (LOEO) approach where the training set was composed of the total number of environments ( nE ) minus one, and the remaining environment was used as testing set, meaning that each environment was used as testing set exactly one time. For this reason, only the average prediction accuracy for each environment was reported since only one-fold (testing set) was obtained for each environment. However, across environments, in addition to the average Pearson correlation, it was also possible to estimate the standard error. The Pearson´s correlation across environments (Global) in LOEO cross-validation was computed averaging the predictions resulting in each of the environments under study in each year. Under this approach the tuning process for the PLS was done exactly as was done under the 7FCV strategy.

## Results

**FIGURE 1:**
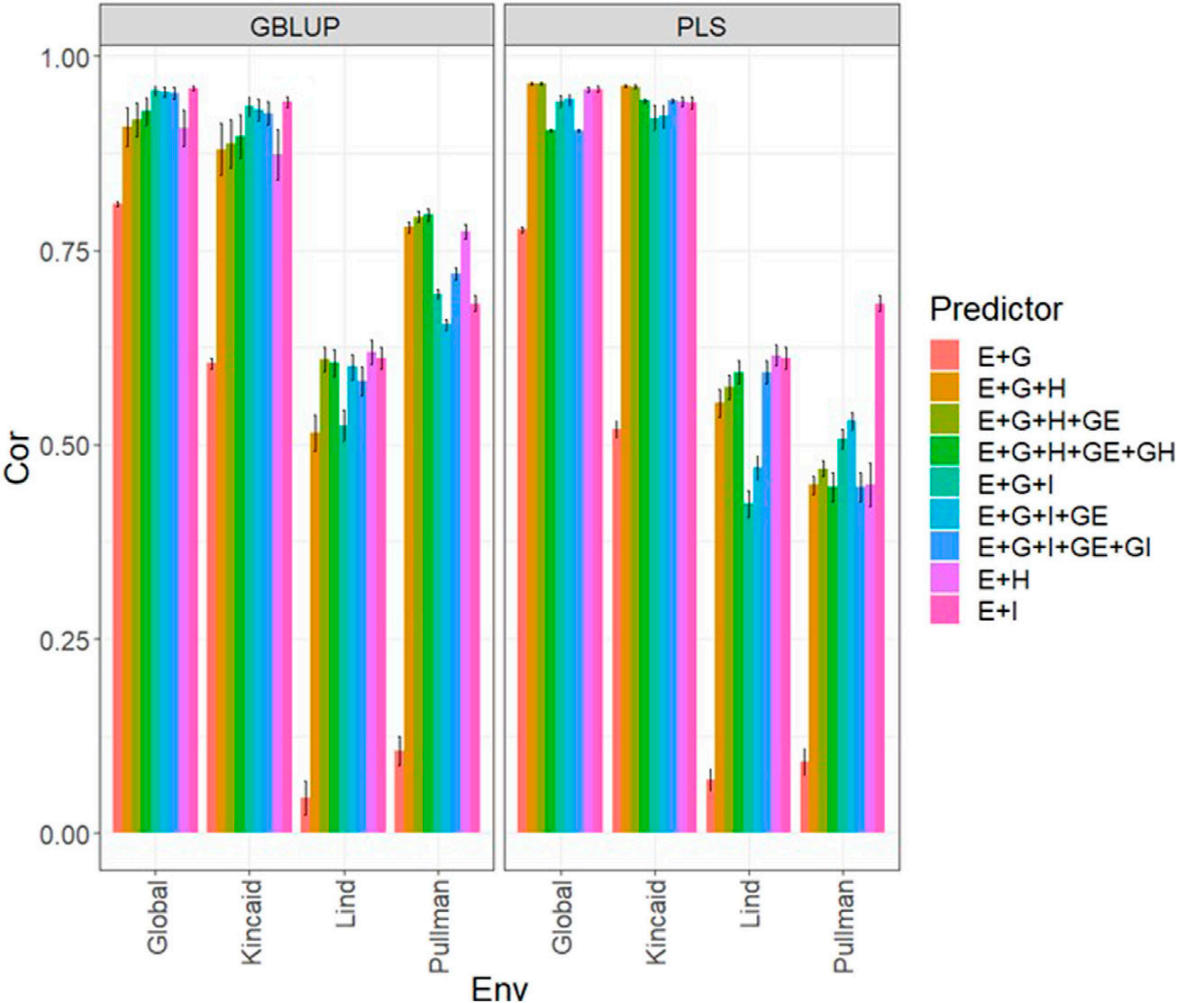
Dataset 1 (year 2019). Pearson´s correlation (Cor) and their corresponding Standard Error (SE) for each location and across location (Global) under tested lines in tested environments (7FCV) for nine evaluated predictors under a GBLUP and PLS models.

**FIGURE 2:**
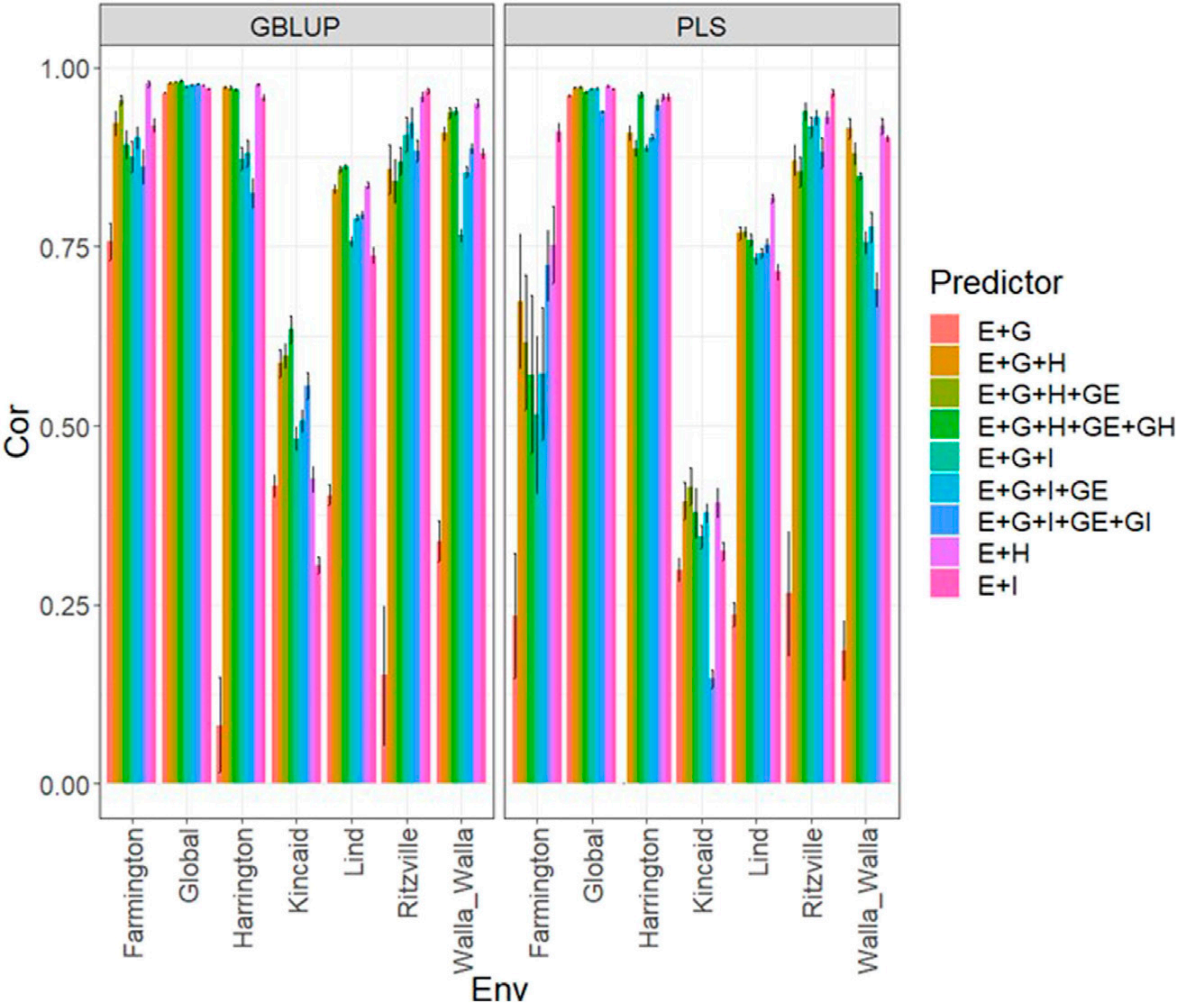
Dataset 2 (year 2020). Pearson´s correlation (Cor) and their corresponding Standard Error (SE) for each location and across location (Global) under tested lines in tested environments (7FCV) for nine evaluated predictors under a GBLUP and PLS models.

**FIGURE 3:**
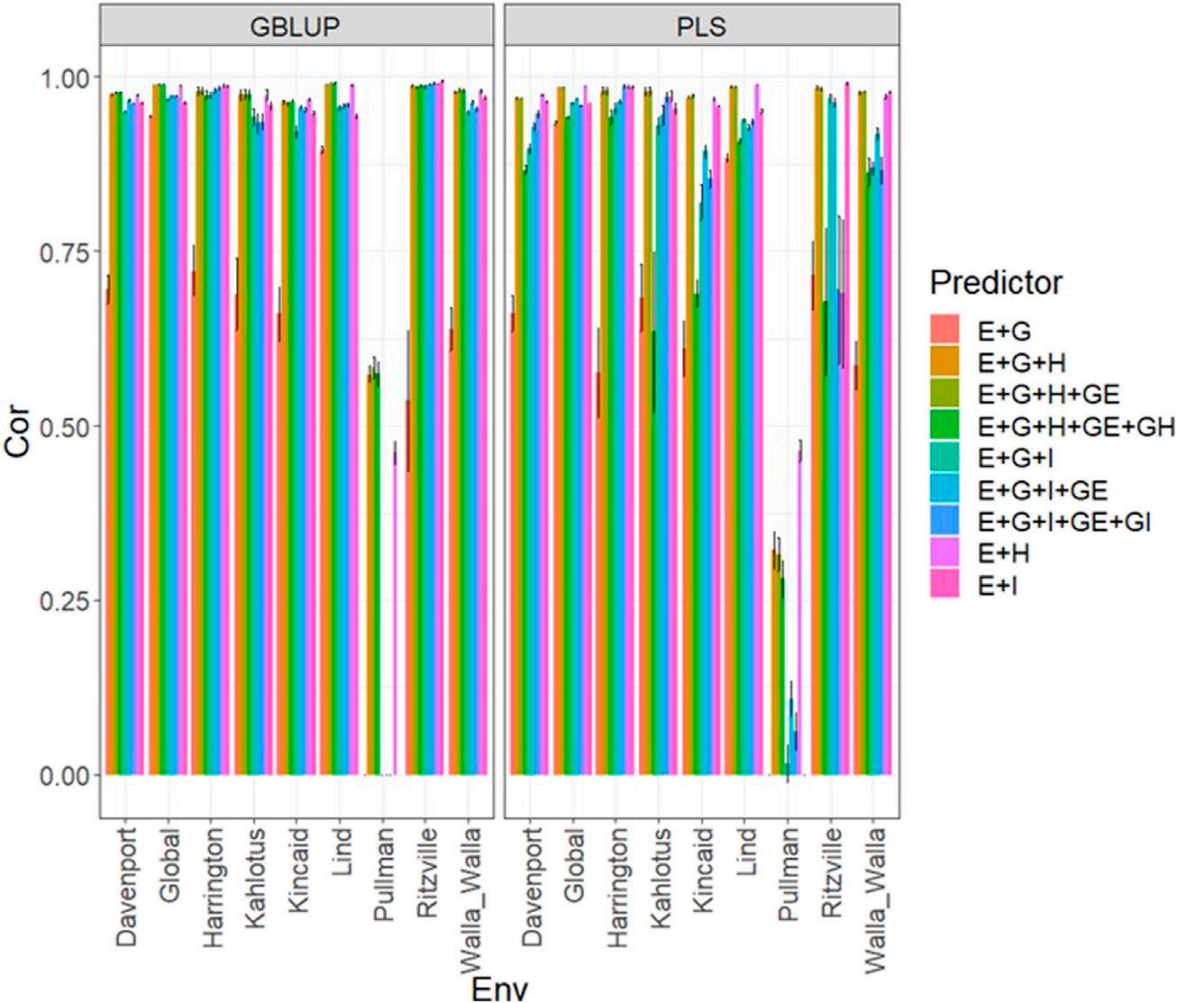
Dataset 3 (year 2021). Pearson´s correlation (Cor) and their corresponding Standard Error (SE) for each location and across location (Global) under tested lines in tested environments (7FCV) for nine evaluated predictors under a GBLUP and PLS models.

**FIGURE 4:**
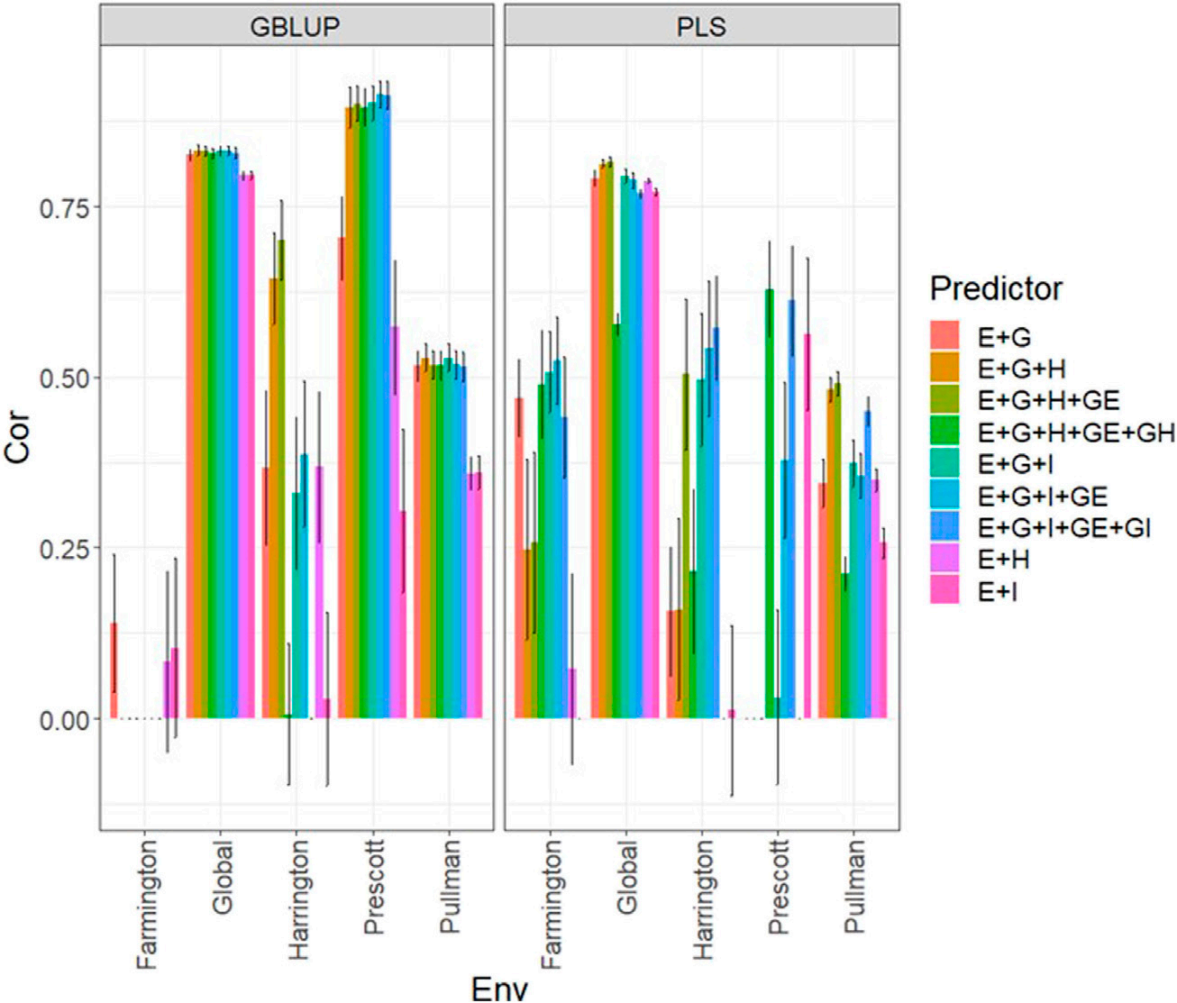
Dataset 4 (year 2022). Pearson´s correlation (Cor) and their corresponding Standard Error (SE) for each location and across location (Global) under tested lines in tested environments (7FCV) for nine evaluated predictors under a GBLUP and PLS models.

**FIGURE 5:**
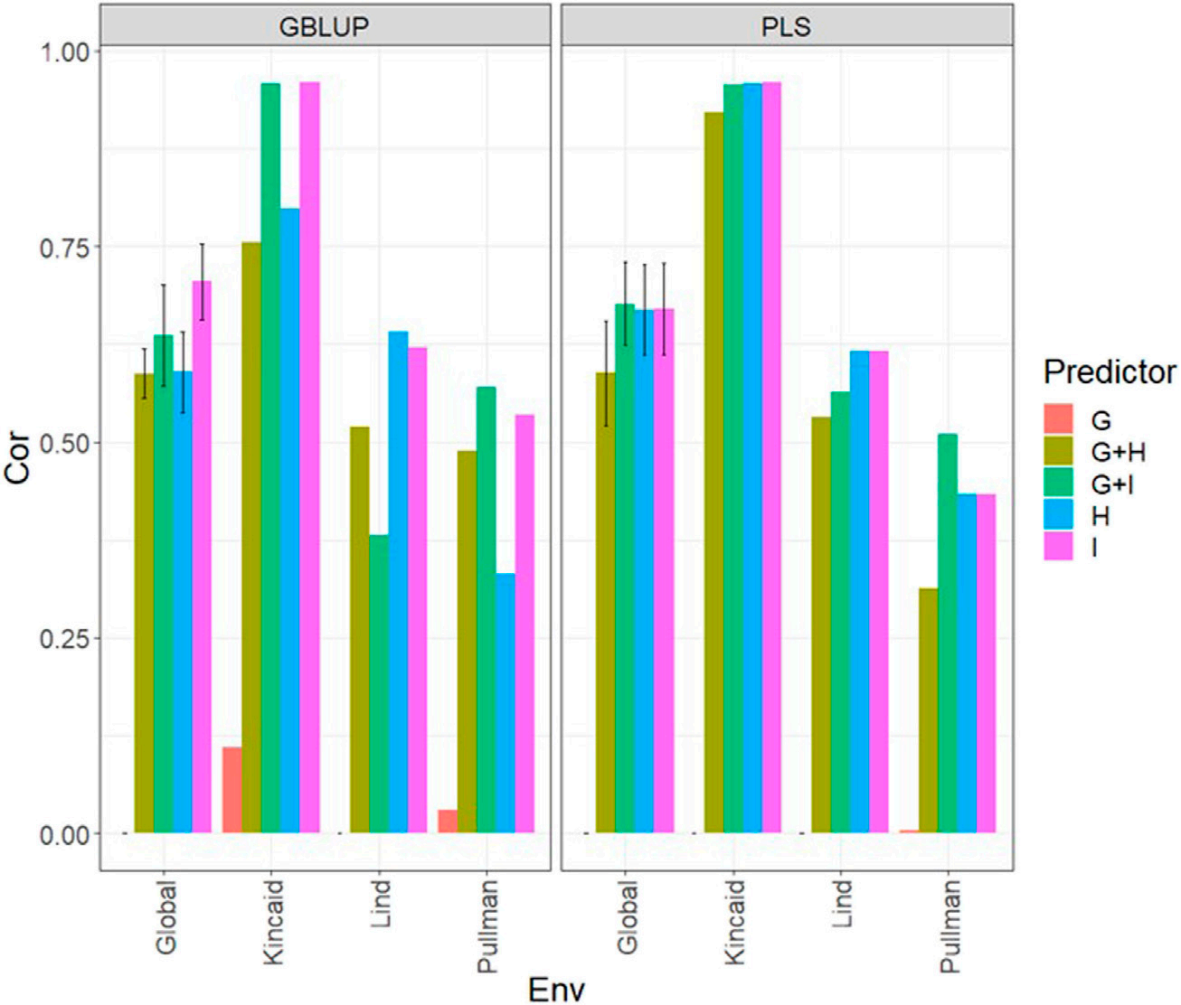
Dataset 1 (year 2019). Pearson´s correlation (Cor) and their corresponding Standard Error (SE) for each location and across location (Global) under tested lines in untested environments (LOEO) for nine evaluated predictors under a GBLUP and PLS models.

**FIGURE 6:**
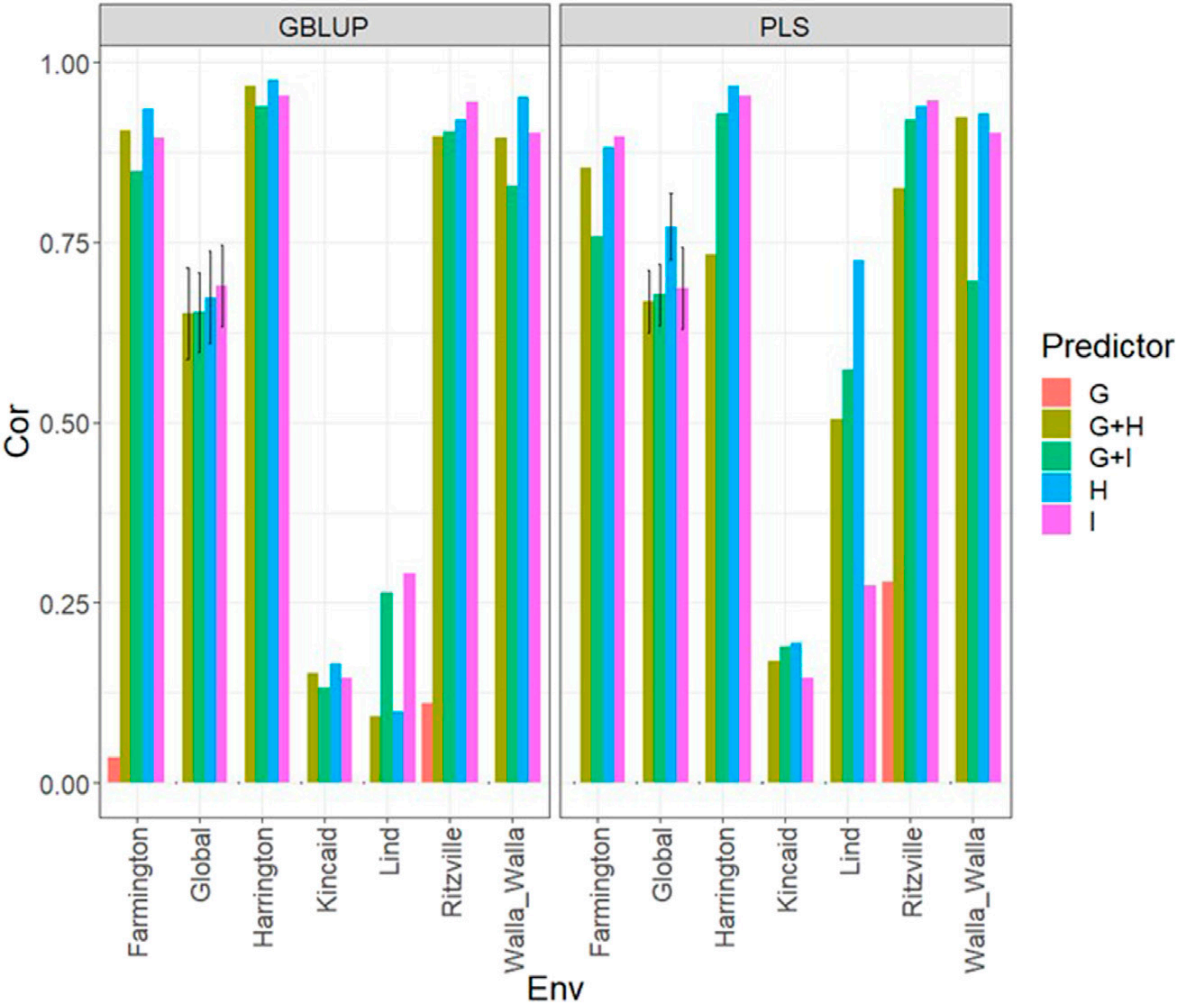
Dataset 2 (year 2020). Pearson´s correlation (Cor) and their corresponding Standard Error (SE) for each location and across location (Global) under tested lines in untested environments (LOEO) for nine evaluated predictors under a GBLUP and PLS models.

**FIGURE 7:**
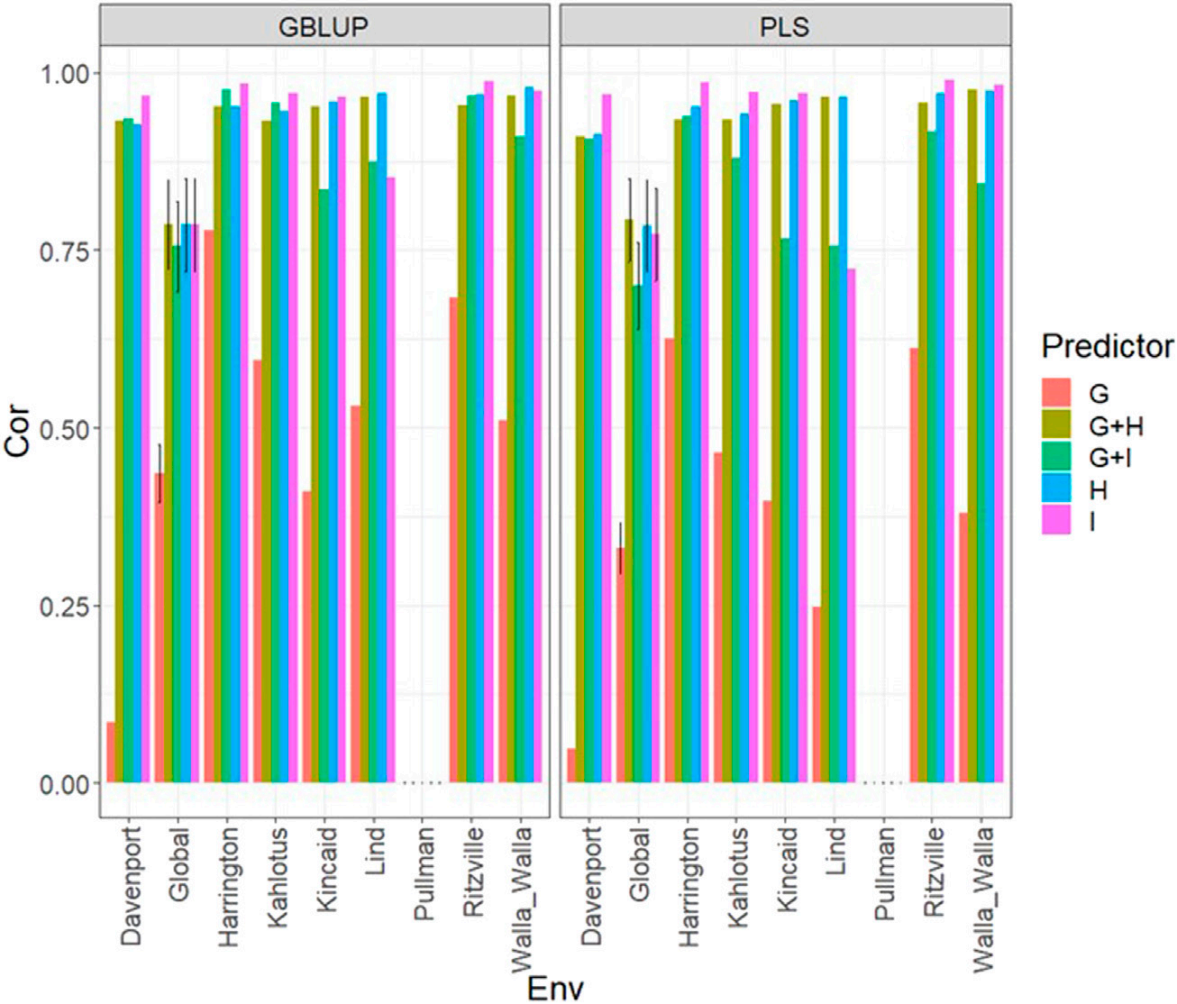
Dataset 3 (year 2021). Pearson´s correlation (Cor) and their corresponding Standard Error (SE) for each location and across location (Global) under tested lines in untested environments (LOEO) for nine evaluated predictors under a GBLUP and PLS models.

**FIGURE 8:**
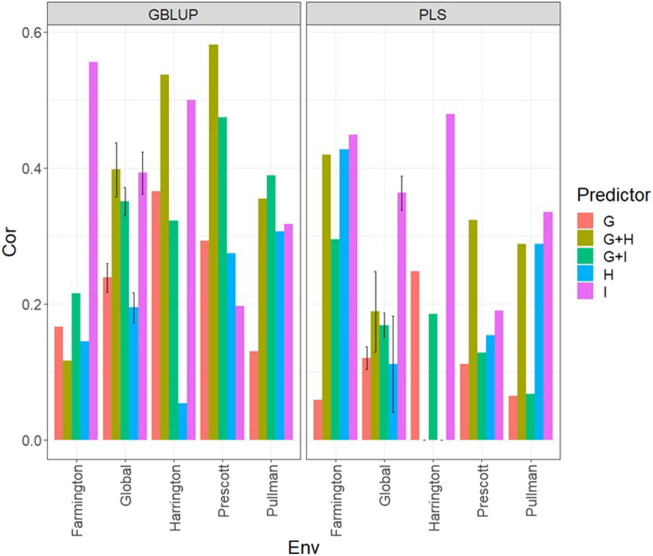
Dataset 4 (year 2022). Pearson´s correlation (Cor) and their corresponding Standard Error (SE) for each location and across location (Global) under tested lines in untested environments (LOEO) for nine evaluated predictors under a GBLUP and PLS models.

The results are provided in three sections. The first section provides, for each data set (each year), the variance component estimates of G, GE, residuals, and heritability. The second section outlines the results under tested lines in tested environments (7FCV) for each data set. The final section highlights the results under tested lines in untested environments for each data set. Supplementary Tables SA2, SA9 contain the results displayed in Figures 1–8.

### Variance components and heritability

We note in Table 2 that the heritabilities of years 2019, 2020 and 2021 are larger than 0.70; however, the heritability for year 2022 is low (0.099). Also, we can see in Table 2 that the GE interaction term is not relevant in years 2019, 2020 and 2022. We note that each of the four data sets is unbalanced since each wheat line was evaluated on average in 1.462, 1.252, 1.711, and 1.309 environments in 2019, 2020, 2021 and 2022, respectively. It is important to note that the environments evaluated in each year were 3, 6, 8, and 6, respectively. It can also be observed in Table 2 that the replications of each line in each environment were less than two since some lines were evaluated in replicated experiments while the remainder were examined in unreplicated experiments.

**TABLE 2:** Heritability estimates (H2) of grain yield (GY) in each of the 4 years and variance components (Vcomp) for the genotypes (G), genotype by environment interaction (GE) and Residual, n_e denotes the average number of locations, n_r denotes the average number of replications of each genotype. Year is the column to differentiate each data set.

Name	Vcomp	Trait	Year	H2	n_r	n_e
GE	0.001	GY	2019	0.867	1.626	1.462
G	1139.850	GY	2019		1.626	1.462
Residual	194.356	GY	2019		1.626	1.462
GE	0.187	GY	2020	0.743	1.569	1.256
G	129.871	GY	2020		1.569	1.256
Residual	56.022	GY	2020		1.569	1.256
GE	42.588	GY	2021	0.706	1.522	1.711
G	136.440	GY	2021		1.522	1.711
Residual	28.475	GY	2021		1.522	1.711
GE	0.002	GY	2022	0.099	1.390	1.309
G	12.080	GY	2022		1.390	1.309
Residual	116.106	GY	2022		1.390	1.309

### Partially tested lines in tested environments

The results under 7FCV for each year are provided. It is important to note that for each model (GBLUP and PLS), nine predictors were evaluated to see how much each part contributes to improve the prediction accuracy and the results are reported for each environment and across environments (Global) for each year.

### Data set 1 (year 2019)

In Figure 1 and Supplementary Table SA2 we observe that under the GBLUP model, the best prediction performance was observed in environment Kincaid and the worst in Lind, while under the PLS model, the best predictions were also observed in the Kincaid environment, and the worst in Pullman. In Figure 1 and Supplementary Table SA2, the worst predictions were observed under the predictor (**E** + **g**). We observed when both types of information are integrated (genomic + multispectral information under its **H** or **I** versions) the best prediction performances were obtained and the predictions across environments and for some environments with Pearson’s correlation values close to one. However, Figure 1 shows that adding the interaction terms **gE**, **gH**, and **gI** to the predictors does not significantly increase prediction performance. Also, it is observed that simple predictors that contain only multispectral information, like **E** + **H** and **E** + **I**, produce similar performance to predictors that incorporate the genotypic information and interaction terms. However, the predictors that integrate both sources of information were more stable and consistent. Regarding the predictions using **H** or **I**, we observe that using **I** information is better since more consistent results than the **H** information and larger Pearson’s correlation are observed. In the models, we observe that both models are effective in this prediction problem and with this data, yet the predictions using GBLUP were better in most cases (Supplementary Table SA2).

### Data set 2 (year 2020)

Under the GBLUP and PLS models, the best predictions were observed in environment Ritzville and the worst in Kincaid; however, very competitive predictions were observed in most environments except for Kincaid (Figure 2; Supplementary Table SA3). Also, the worst predictions were observed under the predictor **E** + **g** (see Figure 2; Supplementary Table SA3). The best prediction performances were observed when both types of information are integrated (genomic + multispectral l information under its **H** or **I** versions) with Pearson’s correlation values close to one in some environments and across environments (Figure 2). Again, we observe in Figure 2; Supplementary Table SA3 that adding interaction terms **gE**, **gH**, and **gI** in the predictors does not provide a relevant increase in performance. In the 2020 data, we observed simple predictors with only multispectral information like **E** + **H** and **E** + **I** produce a similar performance to more complex predictors that incorporate the genotypic information and some interaction terms, but predictors with both sources of information are more stable and consistent. Regarding the predictions using **E** + **H** and **E** + **I**, we observed the predictor **E** + **H** produced results closer to one for Pearson’s correlation values. Both models were effective in this prediction scenario and with this data, but the predictions using GBLUP were better (Supplementary Table SA3).

### Data set 3 (year 2021)

In Figure 3 and Supplementary Table SA4 **,** we can note that under both models (GBLUP and PLS), the worst predictions were observed in Pullman. In the remaining environments, the predictions were effective since they were close to one in terms of Pearson’s correlation. The worst predictions were observed under the predictor **E + g** and the best when both types of information were integrated (genomic + multispectral information under its **H** or **I** versions) with Pearson’s correlation values also close to one. Again, it was observed in Figure 3; Supplementary Table SA4 that adding interaction terms **gE**, **gH**, and **gI** in the predictors did not improve the prediction performance. It was observed that simple predictors with only multispectral information like **E** + **H** and **E** + **I**, provided similar accuracies to predictors with both types of information (genotypic + multispectral information) and interaction terms. However, we observed more stable and consistent predictions in predictors that integrated both sources of information. The predictions using **H** or **I** did not produce relevant differences since, in some cases, using **I** information provided slightly better results than using **H** or *vice versa*. In the models, we observed both models were effective in this prediction scenario, and with this data, the predictions using GBLUP were slightly better (Supplementary Table SA4).

### Data set 4 (year 2022)

In Figure 4 and Supplementary Table SA5, we note that in the GBLUP model, the best and worst predictions were observed in Prescott and Farmington, respectively, while under the PLS model, the environment’s performance showed no difference. However, in both models, the predictions were not as high as those observed in the previous years. Also, the worst predictions were observed with the predictor **E** + **g** (see Figure 4; Supplementary Table SA5) and the best joining of the two types of information (genomic + multispectral information under its **H** or **I** versions). Again, we observed that adding interaction terms **gE**, **gH**, and **gI**, in the predictors did not improve the prediction performance regarding the additive integration of both types of information. Also, we observed that simple predictors with only multispectral information, like **E** + **H** and **E** + **I,** provided more competitive accuracies than predictors with both types of information (genotypic + multispectral information) and interaction terms. Yet we did not observe using the predictor **E** + **H** to give the best predictions. More stable and consistent predictions were observed in predictors that integrated both sources of information. Also, regarding the predictions using **H** or **I**, we observed non-relevant differences between them since sometimes using **I** information provides slightly better results than using **H** or *vice versa*. We observed that both models faced difficulties in predicting some environments but that the GBLUP model outperformed the PLS model (Supplementary Table SA5).

### Partially tested lines in untested environments

In this section, the results under LOEO for each year are reported. For each model (GBLUP and PLS), five predictors were evaluated to see how much each input contributed to the improvement of prediction accuracy in a complete environment. For each year, the results are reported for each environment and across environments (Global).

### Data set 1 (year 2019)

Under the LOEO cross-validation, in both types of models (GBLUP and PLS), we observed the best predictions under Kincaid (Cor>0.75) and the worst under the Lind and Pullman environment with Cor values less than 0.5 (Figure 5; Supplementary Table SA6). The worst predictions were observed only when the genomic information was used (**g**) and the best when both types of information were integrated (Figure 5; Supplementary Table SA6). It was observed that the predictors with only multispectral information (**H** or **I**) provided similar accuracies to predictors with both types of information. Of note, in most environments, the best predictions were observed with the predictors with only multispectral information (**H** and **I**) and within those observations, the best predictions were observed using only the **H** information. The observed models displayed respectable predictions, albeit with considerably lower accuracy than those observed under the 7FCV strategy.

### Data set 2 (year 2020)

Under both types of models (GBLUP and PLS), again under the LOEO cross-validation, we observed the best predictions found under the Farmington, Harrington, Ritzville and Walla Walla (with Cor>0.75 most times) and the worst under the Kincaid and Lind environments, with Cor values less than 0.3 (Figure 6; Supplementary Table SA7). In addition, the worst predictions were observed using only the genomic information (**g**) and the best when only the multispectral information was used, with better performance using **H** in place of **I**, but with no large difference between the two sources of multispectral information. However, very competitive predictions were observed when both types of information were used. We noted good predictions with both models (Cor>0.75) for some environments but modest for others (Cor<0.3).

### Data set 3 (2021)

Under the LOEO cross-validation and both models in this data set, for all environments, it was possible to obtain strong predictions (Cor>0.75 in most cases), with the exception of Pullman, where the predictions in terms of Pearson´s correlation were around 0. Using only the genomic information (**g**) provided the worst predictions and the best predictions were obtained when only the multispectral information was used (Figure 7; Supplementary Table SA8), with better performance using **I** than **H**, but with similar performance between the two sources of multispectral information. However, joining both types of information provided competitive predictions. We observed that both models had strong predictions (Cor>0.75) for many environments, with only one poor result (Cor ≈ 0.0).

### Data set 4 (year 2022)

Under both models and the LOEO cross-validation for all environments, it was not possible to obtain good predictions since, in many environments and some predictors, the predictions in terms of Pearson´s correlation were less than 0.2. But even in this year with lower predictions, the worst performance was obtained using only the genomic information (**g**) and the best when only the multispectral information was used (Figure 8; Supplementary Table SA9), with better performance using **I** than **H**, but with similar performance between the two types of multispectral information. However, joining both types of information provided very competitive predictions. With both models, it was not possible to obtain good predictions (with Cor<0.5) with some predictions lower than 0.2.

## Discussion

As a predictive methodology, GS cannot always guarantee high prediction accuracies since many factors influence its success. For this reason, continuing research to optimize this methodology to lower uncertainty is worthwhile. Adding extra predictors as inputs in the modeling process has been one of the many explored approaches. This approach is very promising since it is becoming more cost-effective to collect extra inputs like omics data (phenomics, proteomics, transcriptomics, etc.) and environmental covariates. Under the assumption that these extra inputs capture complementary information to the available inputs (like genomics information), it can be expected that adding this extra information to the prediction models will improve the prediction accuracy of GS.

We started collecting genomic information in the wheat breeding program in 2016 with the goal of implementing GS within the program. In 2018, we began routinely collecting phenotypic data using UAS technology to provide additional inputs into selection. From our results, it is clear that adding the phenotypic information as inputs enhances the prediction accuracy of GS. However, as expected, the increase in prediction accuracy is larger under the scheme of cross-validation partially tested lines in tested environments and lower for the partially tested lines in untested environments. We found that using only the phenotypic information in this study is much more effective than using only the genomics information. However, combining both sources of information produced the highest prediction accuracies most times. We found that adding the interaction terms of **gE**, **gH**, and **gI** did not improve the prediction accuracies when both sources of information were used. Also, we observed that, in many environments, using only the multispectral information produced the best prediction accuracies.

Under the partially tested lines in tested environments, the prediction accuracies were very high (Cor>0.75), demonstrating that this cross validation is quite safe to use with GS. However, this was not true for all environments, so a deeper understanding of why low prediction accuracies were obtained under these environments is required. However, we know that the time to measure multispectral information is a key factor in enhancing prediction performance, and it is of paramount importance to properly preprocess this multispectral information. More refinements are required under the modeling process and data preprocessing methods to guarantee with a high probability a successful implementation of GS.

Under the partially tested lines in untested environments, the prediction accuracies were lower, with only a few environments having a prediction value greater than 0.75. For example, in 2020 and 2021, most of the environments showed good predictions (Cor>0.75), but in 2019, only one environment reached a strong prediction level (Cor>0.75). These results were not unexpected since this method of cross-validation is complex, but even in this scenario, it was possible to reach good predictions for some environments. Again, the key source of information was the multispectral information.

Our results show that the multispectral information allowed for enhancements to the prediction performance of GS, and most times, even the multispectral information alone produced high prediction accuracies. However, combining multispectral and genomic information, in addition to producing accurate predictions, also helped reduce the variance (which adds stability), which leads us to conclude that integrating other sources of information can help improve the prediction accuracy of GS. Each source of information is discrete but complementary and provides information that is key to capturing all the inputs related to the trait of interest. However, we are aware that adding these extra inputs to the modeling process imposes challenges in the modeling process to avoid the problem of overfitting.

Our results are in agreement with those reported by Rutkoski et al. (2016), Montesinos-López et al. (2017), and Krause et al. (2019) that reported increase in prediction accuracy for grain yield in wheat by using spectral reflectance indices. Also, our findings agree with the study by Royo et al. (2007) that reported reflectance measurements in wheat were the most important predictors for grain yield. Also, our findings are similar to those of Guo et al. (2020) that found an increase in prediction accuracy by integrating secondary traits in the prediction models, concluding that integrating high throughput phenotyping in the modelling process could potentially accelerate selection in wheat.

As noted by Atefi et al. (2021) and supported by our results, autonomous robotic technologies have the potential to substantially increase the capacity, speed, accuracy, and repeatability of data collection in plant phenotyping activities. Many robotic systems have been successfully developed and deployed in greenhouse and field settings and tested on various plant species (corn, wheat, specialty crops, and vineyards). These systems can accurately measure many plant-related characteristics like morphology, structure, development, and physiology (Atefi et al., 2021). Adding these additional phenotypic data into genomic selection models improves prediction accuracy and enhances the selection of new lines in plant breeding programs.

Also, it is important to point out that in general the best predictions were observed under the GBLUP model, even though the PLS model is very useful for prediction problems where the number of independent variables ( p) is larger than the number of observations ( n) and when predictors are highly correlated. In the context of genomic prediction there is evidence that the GBLUP model performs well in most applications and has the advantage of not requiring a time-consuming tuning process. However, the prediction performance observed with the PLS model was very competitive and its tuning process is not difficult since it was only tuned by the number of principals components.

Our results also confirm that there is the potential to increase genetic gain in grain yield by incorporating additional inputs in the prediction models. However, these inputs should be of high quality and related to the predicted trait. Still, there are many problems to implement GS methodology in many applied breeding programs since many factors affect its performance, all of them needing optimization using as many data science tools as possible. Global optimization of all factors that impact prediction accuracy using GS methodology is complex and for the moment each factor is being optimized as a separate problem. However, global optimization is expected to be more efficient. Also, the collection of other ‘omics’ data that integrate in optimal ways can increase the probability that GS can be used as a practical tool in plant breeding programs. Unfortunately, the use of more input data increases the complexity of analysis since more computational resources and sophisticated statistical analyses are required (Lopez-Cruz et al., 2020), but with the advance in computing power and state-of-the-art statistical machine learning algorithms, these difficulties can typically be solved.

## Conclusion

Using wheat data from the Washington State University soft white winter wheat breeding program, we found that integrating high throughput phenotypic and genomic information into prediction models significantly enhances prediction performance, as opposed to using only genomic information. We also observed that using only the phenotypic data, in many cases, produced the best prediction accuracies; however, this finding was not consistently observed. As expected, under the partially tested lines in tested environments, we obtained, in most cases, strong prediction accuracies with both models (GBLUP and PLS), with better performance using GBLUP. Less accurate predictions were observed under the partially tested lines in untested environments, with robust predictions in most 2020 and 2021 environments but low or moderate predictions in 2019 and 2022. Our findings corroborate the importance of phenotypic information to enhance prediction accuracy in GS and emphasize that phenotypic information has significant promise to improve GS by providing better predictions than genomic information alone. We see great potential for improving high throughput phenotypic data collection and processing as well as the overall modeling process in the optimal integration of genomic, phenomic, and other sources of information.

## Data Availability

The datasets presented in this study can be found in online repositories. The names of the repository/repositories and accession number(s) can be found below: https://doi.org/10.7273/000004567.

## References

[B1] Atefi A. Ge Y. Pitla S. Schnable J. (2021). Robotic technologies for high-throughput plant phenotyping: Contemporary reviews and future perspectives. Front. Plant Sci. 12, 611940. 10.3389/fpls.2021.611940 34249028 PMC8267384

[B2] Atkinson J. A. Jackson R. J. Bentley A. R. Ober E. Wells D. M. (2018). “Field phenotyping for the future,” in Annual plant reviews online (Hoboken: Wiley). 10.1002/9781119312994.apr0651

[B3] Bao Y. Tang L. Srinivasan S. Schnable P. S. (2019). Field-based architectural traits characterisation of maize plant using time-of-flight 3D imaging. Biosyst. Eng. 178, 86–101. 10.1016/j.biosystemseng.2018.11.005

[B4] Basnet B. R. Crossa J. Dreisigacker S. Pérez-Rodríguez P. Manes Y. Singh R. P. (2019). Hybrid wheat prediction using genomic, pedigree, and environmental covariables interaction models. Plant Genome 12, 180051. 10.3835/plantgenome2018.07.0051 PMC12810112 30951082

[B5] Bates D. Mächler M. Bolker B. Walker S. (2015). Fitting linear mixed-effects models using lme4. J. Stat. Softw. 67, 1–48. 10.18637/jss.v067.i01

[B6] Baweja H. S. Parhar T. Mirbod O. Nuske S. (2018). StalkNet: A deep learning pipeline for high-throughput measurement of plant stalk count and stalk width bt – field and service robotics. Cham: Springer International Publishing, 271–284.

[B7] Boulesteix A. L. Strimmer K. (2006). Partial least squares: A versatile tool for the analysis of high-dimensional genomic data. Brief. Bioinform 8, 32–44. 10.1093/bib/bbl016 16772269

[B8] Breitzman M. W. Bao Y. Tang L. Schnable P. S. Salas-Fernandez M. G. (2019). Linkage disequilibrium mapping of high-throughput image-derived descriptors of plant architecture traits under field conditions. F. Crop. Res. 244, 107619. 10.1016/j.fcr.2019.107619

[B10] Costa-Neto G. Crossa J. Fritsche-Neto R. (2021b). Enviromic assembly increases accuracy and reduces costs of the genomic prediction for yield plasticity in maize. Front. Plant Sci. 12, 717552. 10.3389/fpls.2021.717552 34691099 PMC8529011

[B11] Costa-Neto G. Fritsche-Neto R. Crossa J. (2021a). Nonlinear kernels, dominance, and envirotyping data increase the accuracy of genome-based prediction in multi-environment trials. Heredity 126, 92–106. 10.1038/s41437-020-00353-1 32855544 PMC7852533

[B12] Crossa J. Fritsche-Neto R. Montesinos-Lopez O. A. Costa-Neto G. Dreisigacker S. Montesinos-Lopez A. (2021). The modern plant breeding triangle: Optimizing the use of genomics, phenomics, and enviromics data. Front. Plant Sci. 12, 651480. 10.3389/fpls.2021.651480 33936136 PMC8085545

[B13] Crossa J. Pérez-Rodríguez P. Cuevas J. Montesinos-López O. A. Jarquín D. de Los Campos G. (2017). Genomic selection in plant breeding: Methods, models, and perspectives. Trends Plant Sci. 22 (11), 961–975. 10.1016/j.tplants.2017.08.011 28965742

[B14] Cuevas J. Montesinos-López O. Juliana P. Guzmán C. Pérez-Rodríguez P. González-Bucio J. (2019). Deep Kernel for genomic and near infrared predictions in multi-environment breeding trials. Genes. genom. Genet. 9, 2913–2924. 10.1534/g3.119.400493 PMC6723142 31289023

[B15] Endelman J. B. (2011). Ridge regression and other kernels for genomic selection with R package rrBLUP. Plant Genome 4, 250–255. 10.3835/plantgenome2011.08.0024

[B16] Fernandez M. G. S. Bao Y. Tang L. Schnable P. S. (2017). A high-throughput, field-based phenotyping technology for tall biomass crops. Plant Physiol. 174, 2008–2022. 10.1104/pp.17.00707 28620124 PMC5543940

[B17] Fischer G. (2009). “World food and agriculture to 2030/50,” in Technical Paper From the Expert Meeting on How to Feed the World in, Rome, Rome, 24-26 June 2009.

[B18] Furbank R. T. Tester M. (2011). Phenomics – technologies to relieve the phenotyping bottleneck. Trends Plant Sci. 16, 635–644. 10.1016/j.tplants.2011.09.005 22074787

[B19] Gitelson A. A. Merzlyak M. N. (1996). Signature analysis of leaf reflectance spectra: Algorithm development for remote sensing of chlorophyll. J. Plant Physiol. 148 (3-4), 494–500. 10.1016/s0176-1617(96)80284-7

[B20] Guo J. Pradhan S. Shahi D. Khan J. Mcbreen J. Bai G. (2020). Increased prediction accuracy using combined genomic information and physiological traits in a soft wheat panel evaluated in multi-environments. Sci. Rep. 10, 7023. 10.1038/s41598-020-63919-3 32341406 PMC7184575

[B21] Hu H. Campbell M. T. Yeats T. H. Zheng X. Runcie D. E. Covarrubias-Pazaran G. (2021). Multi-omics prediction of oat agronomic and seed nutritional traits across environments and in distantly related populations. Theor. Appl. Genet. 134, 4043–4054. 10.1007/s00122-021-03946-4 34643760 PMC8580906

[B22] Huang M. Balimponya E. G. Mgonja E. M. McHale L. K. Luzi-Kihupi A. Guo-Liang Wang G.-L. (2019). Use of genomic selection in breeding rice (Oryza sativa L.) for resistance to rice blast (Magnaporthe oryzae). Mol. Breed. 39, 114. 10.1007/s11032-019-1023-2

[B23] Iqbal F. Lucieer A. Barry K. (2018). Simplified radiometric calibration for UAS-mounted multispectral sensor. Eur. J. Remote Sens. 51 (1), 301–313. 10.1080/22797254.2018.1432293

[B24] Jarquín D. Crossa J. Lacaze X. Du Cheyron P. Daucour J. Lorgeou J. (2014). A reaction norm model for genomic selection using highdimensional genomic and environmental data. Theor. Appl. Genet. 127, 595–607. 10.1007/s00122-013-2243-1 24337101 PMC3931944

[B25] Jarquin D. de Leon N. Romay C. Bohn M. Buckler E. S. Ciampitti I. (2021). Utility of climatic information via combining ability models to improve genomic prediction for yield within the genomes to fields maize project. Front. Genet. 11, 592769. 10.3389/fgene.2020.592769 33763106 PMC7982677

[B26] Jay S. Rabatel G. Hadoux X. Moura D. Gorretta N. (2015). In-field crop row phenotyping from 3D modeling performed using structure from Motion. Comput. Electron. Agric. 110, 70–77. 10.1016/j.compag.2014.09.021

[B27] Kicherer A. Herzog K. Pflanz M. Wieland M. Rüger P. Kecke S. (2015). An automated field phenotyping pipeline for application in grapevine research. Sensors 15, 4823–4836. 10.3390/s150304823 25730485 PMC4435124

[B28] Krause M. R. González-Pérez L. Crossa J. Pérez-Rodríguez P. Montesinos-López O. Singh R. P. (2019). Hyperspectral reflectance derived relationship matrices for genomic prediction of grain yield in wheat. Genes. genom. Genet. 9, 1231–1247. 10.1534/g3.118.200856 PMC6469421 30796086

[B29] Lopes C. M. Graça J. Sastre J. Reyes M. Guzmán R. Braga R. (2016). “Vineyard yeld estimation by VINBOT robot-preliminary results with the white variety Viosinho,” in Proceedings 11th int. Terroir congress. Editors Jones G. Doran N. (Ashland, USA: Southern Oregon University), 458–463.

[B30] Lopez-Cruz M. Olson E. Rovere G. Crossa J. Dreisigacker S. Mondal S. (2020). Regularized selection indices for breeding value prediction using hyperspectral image data. Sci. Rep. 10, 8195. 10.1038/s41598-020-65011-2 32424224 PMC7235263

[B31] Messina C. D. Technow F. Tang T. Totir R. Gho C. Cooper M. (2018). Leveraging biological insight and environmental variation to improve phenotypic prediction: Integrating crop growth models (CGM) with whole genome prediction (WGP). Eur. J. Agron. 100, 151–162. 10.1016/j.eja.2018.01.007

[B32] Meuwissen T. H. E. Hayes B. J. Goddard M. E. (2001). Prediction of total genetic value using genome-wide dense marker maps. Genetics 157, 1819–1829. 10.1093/genetics/157.4.1819 11290733 PMC1461589

[B33] Mevik B.-H. Wehrens R. (2007). The pls package: Principal component and partial least squares regression in R. J. Stat. Softw. 18, 1–24. 10.18637/jss.v018.i02

[B34] Millet E. J. Kruijer W. Coupel-Ledru A. Alvarez Prado S. Cabrera-Bosquet L. Lacube S. (2019). Genomic prediction of maize yield across European environmental conditions. Nat. Genet. 51, 952–956. 10.1038/s41588-019-0414-y 31110353

[B35] Montesinos-López A. Montesinos-López O. A. Cuevas J. Mata-López W. A. Burgueño J. Mondal S. (2017). Genomic Bayesian functional regression models with interactions for predicting wheat grain yield using hyper-spectral image data. Plant Methods 13 (62), 62–29. 10.1186/s13007-017-0212-4 28769997 PMC5530534

[B36] Montesinos-López O. A. Montesinos-López A. Crossa J. (2022). Multivariate statistical machine learning methods for genomic prediction. Switzerland: Springer Nature. ISBN: 978-3-030-89012-4. 36103587

[B37] Monteverde E. Gutierrez L. Blanco P. Pérez de Vida F. Rosas J. E. Bonnecarrère V. (2019). Integrating molecular markers and environmental covariates to interpret genotype by environment interaction in rice (oryza sativa L.) grown in subtropical areas. G3 (Bethesda) 9 (5), 1519–1531. 10.1534/g3.119.400064 30877079 PMC6505146

[B38] Pérez P. de los Campos G. (2014). Bglr: A statistical package for whole genome regression and prediction. Genetics 198 (2), 483–495. 10.1534/genetics.114.164442 25009151 PMC4196607

[B39] Poland J. A. Brown P. J. Sorrells M. E. Jannink J. L. (2012). Development of high-density genetic maps for barley and wheat using a novel two-enzyme genotyping-by-sequencing approach. PLoS One 7, e32253. 10.1371/journal.pone.0032253 22389690 PMC3289635

[B40] Qiu Q. Sun N. Bai H. Wang N. Fan Z. Wang Y. (2019). Field-based high-throughput phenotyping for maize plant using 3D LiDAR point cloud generated with a “Phenomobile. Front. Plant Sci. 10, 554. 10.3389/fpls.2019.00554 31134110 PMC6514377

[B41] R Core Team (2022). R: A language and environment for statistical computing. Vienna: R Foundation for Statistical Computing.

[B42] Rahaman M. M. Chen D. Gillani Z. Klukas C. Chen M. (2015). Advanced phenotyping and phenotype data analysis for the study of plant growth and development. Front. Plant Sci. 6, 619. 10.3389/fpls.2015.00619 26322060 PMC4530591

[B43] Rogers A. R. Dunne J. C. Romay M. C. Bohn M. Buckler E. S. Ciampitti I. A. (2021). The importance of dominance and genotype-by-environment interactions on grain yield variation in a large-scale public cooperative maize experiment. Genes. genom. Genet. 1, jkaa050. 10.1093/g3journal/jkaa050 PMC8022981 33585867

[B44] Rogers A. R. Holland J. B. (2022). Environment-specific genomic prediction ability in maize using environmental covariates depends on environmental similarity to training data. Genes. genom. Genet. 12 (2), jkab440. 10.1093/g3journal/jkab440 PMC9245610 35100364

[B45] Roorkiwal M. Rathore A. Das R. R. Singh M. K. Jain A. Srinivasan S. (2016). Genome-enabled prediction models for yield related traits in Chickpea. Front. Plant Sci. 7, 1666. 10.3389/fpls.2016.01666 27920780 PMC5118446

[B46] Rouse J. W. Jr Haas R. H. Schell J. A. Deering D. W. (1973). Monitoring the vernal advancement and retrogradation (green wave effect) of natural vegetation. No. NASA-CR-132982.

[B47] Royo C. Alvaro F. Martos V. Ramdani A. Isidro J. Villegas D. (2007). Genetic changes in durum wheat yield components and associated traits in Italian and Spanish varieties during the 20th century. Euphytica 155, 259–270. 10.1007/s10681-006-9327-9

[B48] Rutkoski J. Poland J. Mondal S. Autrique E. Pérez L. G. Crossa J. (2016). Canopy temperature and vegetation indices from high-throughput phenotyping improve accuracy of pedigree and genomic selection for grain yield in wheat. Genes. genom. Genet. 6, 2799–2808. 10.1534/g3.116.032888 PMC5015937 27402362

[B49] Sankaran S. Khot L. R. Carter A. H. (2015). Field-based crop phenotyping: Multispectral aerial imaging for evaluation of winter wheat emergence and spring stand. Comput. Electron. Agric. 118, 372–379. 10.1016/j.compag.2015.09.001

[B50] VanRaden P. M. (2008). Efficient methods to compute genomic predictions. J. Dairy Sci. 91 (11), 4414–4423. 10.3168/jds.2007-0980 18946147

[B51] Vázquez-Arellano M. Paraforos D. S. Reiser D. Garrido-Izard M. Griepentrog H. W. (2018). Determination of stem position and height of reconstructed maize plants using a time-of-flight camera. Comput. Electron. Agric. 154, 276–288. 10.1016/j.compag.2018.09.006

[B52] Vidoni R. Gallo R. Ristorto G. Carabin G. Mazzetto F. Scalera L. (2017). “ByeLab: An agricultural mobile robot prototype for proximal sensing and precision farming,” in ASME international mechanical engineering congress and exposition (New York, NY: American Society of Mechanical Engineers), 962.

[B53] Vijayarangan S. Sodhi P. Kini P. Bourne J. Du S. Sun H. (2018). “High-throughput robotic phenotyping of energy sorghum crops BT - field and service robotics,” in Proceedings of 11th International Conference on Field and Service Robotics (FSR '17), Zurich, Switzerland, 12-15 September 2017, 99–113.

[B54] Washburn J. D. Cimen E. Ramstein G. Reeves T. O'Briant P. McLean G. (2021). Predicting phenotypes from genetic, environment, management, and historical data using CNNs. Theor. Appl. Genet. 134 (12), 3997–4011. 10.1007/s00122-021-03943-7 34448888

[B55] Wold S. (2001). Personal memories of the early PLS development. Chemomet. Intel. Lab. Syst. 58, 83–84. 10.1016/S0169-7439(01)00152-6

[B56] Wolfe M. D. Del Carpio D. P. Alabi O. Ezenwaka L. C. Ikeogu U. N. Kayondo I. S. (2017). Prospects for genomic selection in cassava breeding. Plant Genome 10, 15. 10.3835/plantgenome2017.03.0015 PMC7822052 29293806

[B57] Wu P. Y. Stich B. Weisweiler M. Shrestha A. Erban A. Westhoff P. (2022). Improvement of prediction ability by integrating multi-omic datasets in barley. BMC Genomics 23 (1), 200. 10.1186/s12864-022-08337-7 35279073 PMC8917753

[B58] Xu R. Li C. Mohammadpour Velni J. (2018). Development of an autonomous ground robot for field high throughput phenotyping. IFAC Pap. 51, 70–74. 10.1016/j.ifacol.2018.08.063

[B59] Yoosefzadeh-Najafabadi M. Rajcan I. Eskandari M. (2022). Optimizing genomic selection in soybean: An important improvement in agricultural genomics. Heliyon 8, e11873. 10.1016/j.heliyon.2022.e11873 36468106 PMC9713349

[B60] Young S. N. Kayacan E. Peschel J. M. (2019). Design and field evaluation of a ground robot for high-throughput phenotyping of energy sorghum. Precis. Agric. 20, 697–722. 10.1007/s11119-018-9601-6

[B61] Yuan H. Wang N. Bennett R. Burditt D. Cannon A. Chamberlin K. (2018). Development of a ground-based peanut canopy phenotyping system. IFAC Pap. 51, 162–165. 10.1016/j.ifacol.2018.08.081

[B62] Zhang Z. Kayacan E. Thompson B. Chowdhary G. (2020). High precision control and deep learning-based corn stand counting algorithms for agricultural robot. Auton. Robots 44, 1289–1302. 10.1007/s10514-020-09915-y

