# Supplementary material for: Genomics combined with UAS data enhances prediction of grain yield in winter wheat
Source: Front Genet. 2023 Mar 29;14:1124218. doi: 10.3389/fgene.2023.1124218 (PMC10090417; doi:10.3389/fgene.2023.1124218)
Supplement: Supplementary file 1 [file Table1.docx]

**Genomics combined with UAS data enhances prediction of grain yield in winter wheat**

Osval A. Montesinos-López^1^, Andrew W. Herr^2^, José Crossa^3,4^, Arron H. Carter^2*^,

**Appendix**

**Table A1**. Predictors implemented (ETA) under the Bayesian GBLUP model. These nine predictors differentiate between then since some include information only some main effects of environments, genotypes and multispectral information, while others in addition to main effects also take into account some interaction effects.

| ID | ETA |
| --- | --- |
| ETA1 | $E_{i}+g_{j}$; $\boldsymbol{E}=\left( E_{1},\ldots,E_{nE} \right)^{T}\sim N_{I}\left( \boldsymbol{0},\sigma_{E}^{2}\boldsymbol{I} \right)$, for $i=$1,…,$nE$; $\boldsymbol{g}=\left( g_{1},\ldots,g_{J} \right)^{T}\sim N_{J}\left( \boldsymbol{0},\sigma_{g}^{2}\text{G} \right)$, j$=$1,…,$J$ and $\text{G}$ is a genomic relationship matrix as proposed by VanRaden (2008). In matrix notation this predictor is equal to $\boldsymbol{E}+\boldsymbol{g}$**.** |
| ETA2 | $E_{i}+g_{j}+\boldsymbol{H}_{ij};\boldsymbol{H}_{ij}=[H_{ij1,}\ldots, H_{ij10}]$ is a vector of length eleven of multispectral information for the j*th* line and i*th* environment. In matrix notation this predictor is equal to $\boldsymbol{E}+\boldsymbol{g+H}$**.** |
| ETA3 | $E_{i}+g_{j}+\boldsymbol{H}_{ij}+gE_{ij}$; $\boldsymbol{gE=}\left( gE_{11},\ldots,gE_{1J},\ldots,gE_{nEJ} \right)^{T}{\sim N}_{IJ}[\boldsymbol{0},\sigma_{gL}^{2}(\boldsymbol{G}_{GE})]$; with ${\boldsymbol{G}_{GE}\boldsymbol{=Z}}_{E}\boldsymbol{Z}_{E}^{T}⨀\boldsymbol{Z}_{g}\text{G}\boldsymbol{Z}_{g}^{T}$. In matrix notation this predictor is equal to $\boldsymbol{E}+\boldsymbol{g+H+gE}$**.** |
| ETA4 | $E_{i}+g_{j}+\boldsymbol{H}_{ij}+gE_{ij}+g\boldsymbol{H}_{ij}$; $\boldsymbol{g}\boldsymbol{H}_{ij}$ is the interaction term between the genotypes and the multispectral bands. In matrix notation this predictor is equal to $\boldsymbol{E}+\boldsymbol{g+H+gE+gH.}$ |
| ETA5 | $E_{i}+g_{j}+\boldsymbol{I}_{ij}$; $\boldsymbol{I}_{ij}=[I_{ij1,}I_{ij2}, I_{ij3}]$ is a vector of three indices computed from the multispectral information for the j*th* line and i*th* environment. In matrix notation this predictor is equal to $\boldsymbol{E}+\boldsymbol{g+}\boldsymbol{I}$**.** |
| ETA6 | $E_{i}+g_{j}+\boldsymbol{I}_{ij}+gE_{ij}$. In matrix notation this predictor is equal to $\boldsymbol{E}+\boldsymbol{g+}\boldsymbol{I}$**+gI.** |
| ETA7 | $E_{i}+g_{j}+\boldsymbol{I}_{ij}+gE_{ij}+g\boldsymbol{I}_{ij}$; $\boldsymbol{g}\boldsymbol{I}_{ij}$ is the interaction term between the genotypes and the three indices. In matrix notation this predictor is equal to $\boldsymbol{E}+\boldsymbol{g+}\boldsymbol{I}$**+gE+gI.** |
| ETA8 | $E_{i}+\boldsymbol{H}_{ij}$. In matrix notation this predictor is equal to $\boldsymbol{E}+H.$ |
| ETA9 | $E_{i}+\boldsymbol{I}_{ij}. In matrix notation this predictor is equal to \boldsymbol{E}+I.$ |

**Table A2.** Prediction performance for every environment and across environments (Global) of **dataset 1 (Year 2019)** in terms of Pearson´s correlation (Cor) for partially tested lines in tested environments (7FCV). Cor_GBLUP denotes the Cor computed under the GBLUP model, Cor_GBLUP_SE denotes its corresponding standard error. Cor_PLS denotes the Cor computed under the PLS model, Cor_PLS_SE denotes its corresponding standard error.

| CV | Predictor | Year | Env | Cor_GBLUP | Cor_GBLUP_SE | Cor_PLS | Cor_PLS_SE |
| --- | --- | --- | --- | --- | --- | --- | --- |
| 7FCV | E+G | 2019 | Kincaid | 0.605 | 0.019 | 0.520 | 0.031 |
| 7FCV | E+G | 2019 | Lind | 0.046 | 0.058 | 0.068 | 0.038 |
| 7FCV | E+G | 2019 | Pullman | 0.106 | 0.049 | 0.092 | 0.046 |
| 7FCV | E+G | 2019 | Global | 0.810 | 0.008 | 0.777 | 0.010 |
| 7FCV | E+G+H | 2019 | Kincaid | 0.880 | 0.087 | 0.962 | 0.005 |
| 7FCV | E+G+H | 2019 | Lind | 0.515 | 0.060 | 0.554 | 0.048 |
| 7FCV | E+G+H | 2019 | Pullman | 0.780 | 0.019 | 0.449 | 0.031 |
| 7FCV | E+G+H | 2019 | Global | 0.909 | 0.065 | 0.965 | 0.003 |
| 7FCV | E+G+H+GE | 2019 | Kincaid | 0.887 | 0.082 | 0.961 | 0.006 |
| 7FCV | E+G+H+GE | 2019 | Lind | 0.610 | 0.042 | 0.574 | 0.041 |
| 7FCV | E+G+H+GE | 2019 | Pullman | 0.793 | 0.019 | 0.470 | 0.029 |
| 7FCV | E+G+H+GE | 2019 | Global | 0.919 | 0.058 | 0.965 | 0.003 |
| 7FCV | E+G+H+GE+GH | 2019 | Kincaid | 0.897 | 0.072 | 0.943 | 0.007 |
| 7FCV | E+G+H+GE+GH | 2019 | Lind | 0.606 | 0.046 | 0.593 | 0.040 |
| 7FCV | E+G+H+GE+GH | 2019 | Pullman | 0.796 | 0.020 | 0.445 | 0.049 |
| 7FCV | E+G+H+GE+GH | 2019 | Global | 0.929 | 0.048 | 0.905 | 0.005 |
| 7FCV | E+G+I | 2019 | Kincaid | 0.935 | 0.032 | 0.921 | 0.042 |
| 7FCV | E+G+I | 2019 | Lind | 0.525 | 0.053 | 0.424 | 0.045 |
| 7FCV | E+G+I | 2019 | Pullman | 0.694 | 0.016 | 0.508 | 0.033 |
| 7FCV | E+G+I | 2019 | Global | 0.956 | 0.015 | 0.942 | 0.022 |
| 7FCV | E+G+I+GE | 2019 | Kincaid | 0.931 | 0.037 | 0.923 | 0.039 |
| 7FCV | E+G+I+GE | 2019 | Lind | 0.600 | 0.045 | 0.470 | 0.039 |
| 7FCV | E+G+I+GE | 2019 | Pullman | 0.654 | 0.020 | 0.530 | 0.030 |
| 7FCV | E+G+I+GE | 2019 | Global | 0.954 | 0.018 | 0.944 | 0.020 |
| 7FCV | E+G+I+GE+GI | 2019 | Kincaid | 0.927 | 0.040 | 0.943 | 0.007 |
| 7FCV | E+G+I+GE+GI | 2019 | Lind | 0.582 | 0.049 | 0.593 | 0.040 |
| 7FCV | E+G+I+GE+GI | 2019 | Pullman | 0.720 | 0.019 | 0.445 | 0.049 |
| 7FCV | E+G+I+GE+GI | 2019 | Global | 0.953 | 0.020 | 0.905 | 0.005 |
| 7FCV | E+H | 2019 | Kincaid | 0.874 | 0.085 | 0.942 | 0.018 |
| 7FCV | E+H | 2019 | Lind | 0.619 | 0.040 | 0.615 | 0.037 |
| 7FCV | E+H | 2019 | Pullman | 0.774 | 0.025 | 0.449 | 0.073 |
| 7FCV | E+H | 2019 | Global | 0.907 | 0.063 | 0.957 | 0.007 |
| 7FCV | E+I | 2019 | Kincaid | 0.941 | 0.019 | 0.940 | 0.020 |
| 7FCV | E+I | 2019 | Lind | 0.612 | 0.036 | 0.611 | 0.036 |
| 7FCV | E+I | 2019 | Pullman | 0.682 | 0.027 | 0.682 | 0.027 |
| 7FCV | E+I | 2019 | Global | 0.959 | 0.008 | 0.958 | 0.009 |

**Table A3.** Prediction performance for every environment and across environments (Global) of **dataset 2 (Year 2020)** in terms of Pearson´s correlation (Cor) for partially tested lines in tested environments (7FCV). Cor_GBLUP denotes the Cor computed under the GBLUP model, Cor_GBLUP_SE denotes its corresponding standard error. Cor_PLS denotes the Cor computed under the PLS model, Cor_PLS_SE denotes its corresponding standard error.

| CV | Predictor | Year | Env | Cor_GBLUP | Cor_GBLUP_SE | Cor_PLS | Cor_PLS_SE |
| --- | --- | --- | --- | --- | --- | --- | --- |
| 7FCV | E+G | 2020 | Farmington | 0.757 | 0.071 | 0.234 | 0.232 |
| 7FCV | E+G | 2020 | Harrington | 0.082 | 0.177 | 0.000 | 0.000 |
| 7FCV | E+G | 2020 | Kincaid | 0.416 | 0.041 | 0.299 | 0.043 |
| 7FCV | E+G | 2020 | Lind | 0.403 | 0.038 | 0.236 | 0.046 |
| 7FCV | E+G | 2020 | Ritzville | 0.151 | 0.256 | 0.266 | 0.231 |
| 7FCV | E+G | 2020 | Walla_Walla | 0.339 | 0.074 | 0.186 | 0.110 |
| 7FCV | E+G | 2020 | Global | 0.965 | 0.003 | 0.961 | 0.003 |
| 7FCV | E+G+H | 2020 | Farmington | 0.923 | 0.045 | 0.674 | 0.246 |
| 7FCV | E+G+H | 2020 | Harrington | 0.973 | 0.006 | 0.909 | 0.027 |
| 7FCV | E+G+H | 2020 | Kincaid | 0.587 | 0.049 | 0.395 | 0.069 |
| 7FCV | E+G+H | 2020 | Lind | 0.830 | 0.015 | 0.769 | 0.025 |
| 7FCV | E+G+H | 2020 | Ritzville | 0.858 | 0.090 | 0.871 | 0.055 |
| 7FCV | E+G+H | 2020 | Walla_Walla | 0.909 | 0.024 | 0.916 | 0.038 |
| 7FCV | E+G+H | 2020 | Global | 0.979 | 0.003 | 0.972 | 0.004 |
| 7FCV | E+G+H+GE | 2020 | Farmington | 0.955 | 0.018 | 0.616 | 0.249 |
| 7FCV | E+G+H+GE | 2020 | Harrington | 0.972 | 0.007 | 0.887 | 0.029 |
| 7FCV | E+G+H+GE | 2020 | Kincaid | 0.598 | 0.046 | 0.415 | 0.067 |
| 7FCV | E+G+H+GE | 2020 | Lind | 0.858 | 0.010 | 0.770 | 0.018 |
| 7FCV | E+G+H+GE | 2020 | Ritzville | 0.841 | 0.080 | 0.855 | 0.056 |
| 7FCV | E+G+H+GE | 2020 | Walla_Walla | 0.937 | 0.019 | 0.880 | 0.039 |
| 7FCV | E+G+H+GE | 2020 | Global | 0.980 | 0.003 | 0.973 | 0.004 |
| 7FCV | E+G+H+GE+GH | 2020 | Farmington | 0.893 | 0.052 | 0.572 | 0.293 |
| 7FCV | E+G+H+GE+GH | 2020 | Harrington | 0.970 | 0.006 | 0.962 | 0.011 |
| 7FCV | E+G+H+GE+GH | 2020 | Kincaid | 0.634 | 0.052 | 0.378 | 0.092 |
| 7FCV | E+G+H+GE+GH | 2020 | Lind | 0.862 | 0.009 | 0.760 | 0.022 |
| 7FCV | E+G+H+GE+GH | 2020 | Ritzville | 0.869 | 0.052 | 0.939 | 0.030 |
| 7FCV | E+G+H+GE+GH | 2020 | Walla_Walla | 0.940 | 0.014 | 0.848 | 0.015 |
| 7FCV | E+G+H+GE+GH | 2020 | Global | 0.981 | 0.003 | 0.966 | 0.004 |
| 7FCV | E+G+I | 2020 | Farmington | 0.875 | 0.059 | 0.516 | 0.290 |
| 7FCV | E+G+I | 2020 | Harrington | 0.873 | 0.043 | 0.888 | 0.012 |
| 7FCV | E+G+I | 2020 | Kincaid | 0.482 | 0.041 | 0.345 | 0.043 |
| 7FCV | E+G+I | 2020 | Lind | 0.758 | 0.018 | 0.734 | 0.020 |
| 7FCV | E+G+I | 2020 | Ritzville | 0.906 | 0.066 | 0.917 | 0.037 |
| 7FCV | E+G+I | 2020 | Walla_Walla | 0.766 | 0.024 | 0.756 | 0.041 |
| 7FCV | E+G+I | 2020 | Global | 0.974 | 0.003 | 0.970 | 0.002 |
| 7FCV | E+G+I+GE | 2020 | Farmington | 0.902 | 0.038 | 0.573 | 0.244 |
| 7FCV | E+G+I+GE | 2020 | Harrington | 0.881 | 0.048 | 0.903 | 0.012 |
| 7FCV | E+G+I+GE | 2020 | Kincaid | 0.507 | 0.040 | 0.378 | 0.033 |
| 7FCV | E+G+I+GE | 2020 | Lind | 0.790 | 0.013 | 0.741 | 0.018 |
| 7FCV | E+G+I+GE | 2020 | Ritzville | 0.923 | 0.056 | 0.931 | 0.029 |
| 7FCV | E+G+I+GE | 2020 | Walla_Walla | 0.854 | 0.020 | 0.777 | 0.056 |
| 7FCV | E+G+I+GE | 2020 | Global | 0.976 | 0.003 | 0.971 | 0.003 |
| 7FCV | E+G+I+GE+GI | 2020 | Farmington | 0.862 | 0.062 | 0.724 | 0.130 |
| 7FCV | E+G+I+GE+GI | 2020 | Harrington | 0.825 | 0.053 | 0.949 | 0.020 |
| 7FCV | E+G+I+GE+GI | 2020 | Kincaid | 0.556 | 0.049 | 0.146 | 0.033 |
| 7FCV | E+G+I+GE+GI | 2020 | Lind | 0.795 | 0.014 | 0.752 | 0.024 |
| 7FCV | E+G+I+GE+GI | 2020 | Ritzville | 0.884 | 0.040 | 0.882 | 0.056 |
| 7FCV | E+G+I+GE+GI | 2020 | Walla_Walla | 0.887 | 0.017 | 0.691 | 0.064 |
| 7FCV | E+G+I+GE+GI | 2020 | Global | 0.977 | 0.003 | 0.939 | 0.004 |
| 7FCV | E+H | 2020 | Farmington | 0.978 | 0.011 | 0.753 | 0.142 |
| 7FCV | E+H | 2020 | Harrington | 0.976 | 0.006 | 0.959 | 0.013 |
| 7FCV | E+H | 2020 | Kincaid | 0.426 | 0.046 | 0.392 | 0.054 |
| 7FCV | E+H | 2020 | Lind | 0.836 | 0.013 | 0.818 | 0.015 |
| 7FCV | E+H | 2020 | Ritzville | 0.959 | 0.017 | 0.930 | 0.023 |
| 7FCV | E+H | 2020 | Walla_Walla | 0.950 | 0.016 | 0.919 | 0.029 |
| 7FCV | E+H | 2020 | Global | 0.976 | 0.003 | 0.975 | 0.003 |
| 7FCV | E+I | 2020 | Farmington | 0.920 | 0.026 | 0.911 | 0.034 |
| 7FCV | E+I | 2020 | Harrington | 0.959 | 0.012 | 0.960 | 0.012 |
| 7FCV | E+I | 2020 | Kincaid | 0.305 | 0.031 | 0.325 | 0.034 |
| 7FCV | E+I | 2020 | Lind | 0.738 | 0.027 | 0.715 | 0.029 |
| 7FCV | E+I | 2020 | Ritzville | 0.967 | 0.013 | 0.965 | 0.013 |
| 7FCV | E+I | 2020 | Walla_Walla | 0.881 | 0.018 | 0.902 | 0.013 |
| 7FCV | E+I | 2020 | Global | 0.971 | 0.003 | 0.970 | 0.003 |

**Table A4.** Prediction performance for every environment and across environments (Global) of **dataset 3 (Year 2021)** in terms of Pearson´s correlation (Cor) for partially tested lines in tested environments (7FCV). Cor_GBLUP denotes the Cor computed under the GBLUP model, Cor_GBLUP_SE denotes its corresponding standard error. Cor_PLS denotes the Cor computed under the PLS model, Cor_PLS_SE denotes its corresponding standard error.

| CV | Predictor | Year | Env | Cor_GBLUP | Cor_GBLUP_SE | Cor_PLS | Cor_PLS_SE |
| --- | --- | --- | --- | --- | --- | --- | --- |
| 7FCV | E+G | 2021 | Davenport | 0.696 | 0.056 | 0.661 | 0.069 |
| 7FCV | E+G | 2021 | Harrington | 0.722 | 0.099 | 0.576 | 0.169 |
| 7FCV | E+G | 2021 | Kahlotus | 0.689 | 0.137 | 0.683 | 0.126 |
| 7FCV | E+G | 2021 | Kincaid | 0.660 | 0.104 | 0.610 | 0.105 |
| 7FCV | E+G | 2021 | Lind | 0.896 | 0.013 | 0.884 | 0.019 |
| 7FCV | E+G | 2021 | Pullman | 0.000 | 0.000 | 0.000 | 0.000 |
| 7FCV | E+G | 2021 | Ritzville | 0.536 | 0.267 | 0.715 | 0.129 |
| 7FCV | E+G | 2021 | Walla_Walla | 0.639 | 0.081 | 0.586 | 0.094 |
| 7FCV | E+G | 2021 | Global | 0.944 | 0.004 | 0.934 | 0.005 |
| 7FCV | E+G+H | 2021 | Davenport | 0.975 | 0.004 | 0.970 | 0.003 |
| 7FCV | E+G+H | 2021 | Harrington | 0.979 | 0.015 | 0.980 | 0.013 |
| 7FCV | E+G+H | 2021 | Kahlotus | 0.974 | 0.019 | 0.979 | 0.016 |
| 7FCV | E+G+H | 2021 | Kincaid | 0.964 | 0.007 | 0.970 | 0.007 |
| 7FCV | E+G+H | 2021 | Lind | 0.989 | 0.002 | 0.986 | 0.002 |
| 7FCV | E+G+H | 2021 | Pullman | 0.573 | 0.035 | 0.322 | 0.071 |
| 7FCV | E+G+H | 2021 | Ritzville | 0.987 | 0.004 | 0.984 | 0.007 |
| 7FCV | E+G+H | 2021 | Walla_Walla | 0.978 | 0.005 | 0.978 | 0.006 |
| 7FCV | E+G+H | 2021 | Global | 0.989 | 0.001 | 0.985 | 0.001 |
| 7FCV | E+G+H+GE | 2021 | Davenport | 0.977 | 0.004 | 0.969 | 0.003 |
| 7FCV | E+G+H+GE | 2021 | Harrington | 0.979 | 0.016 | 0.980 | 0.013 |
| 7FCV | E+G+H+GE | 2021 | Kahlotus | 0.975 | 0.019 | 0.980 | 0.015 |
| 7FCV | E+G+H+GE | 2021 | Kincaid | 0.962 | 0.007 | 0.973 | 0.005 |
| 7FCV | E+G+H+GE | 2021 | Lind | 0.991 | 0.002 | 0.986 | 0.002 |
| 7FCV | E+G+H+GE | 2021 | Pullman | 0.583 | 0.041 | 0.316 | 0.063 |
| 7FCV | E+G+H+GE | 2021 | Ritzville | 0.985 | 0.005 | 0.983 | 0.007 |
| 7FCV | E+G+H+GE | 2021 | Walla_Walla | 0.981 | 0.006 | 0.978 | 0.006 |
| 7FCV | E+G+H+GE | 2021 | Global | 0.990 | 0.001 | 0.985 | 0.001 |
| 7FCV | E+G+H+GE+GH | 2021 | Davenport | 0.978 | 0.004 | 0.867 | 0.016 |
| 7FCV | E+G+H+GE+GH | 2021 | Harrington | 0.974 | 0.017 | 0.942 | 0.027 |
| 7FCV | E+G+H+GE+GH | 2021 | Kahlotus | 0.975 | 0.020 | 0.634 | 0.303 |
| 7FCV | E+G+H+GE+GH | 2021 | Kincaid | 0.965 | 0.007 | 0.689 | 0.052 |
| 7FCV | E+G+H+GE+GH | 2021 | Lind | 0.991 | 0.002 | 0.907 | 0.010 |
| 7FCV | E+G+H+GE+GH | 2021 | Pullman | 0.575 | 0.045 | 0.280 | 0.072 |
| 7FCV | E+G+H+GE+GH | 2021 | Ritzville | 0.986 | 0.004 | 0.678 | 0.280 |
| 7FCV | E+G+H+GE+GH | 2021 | Walla_Walla | 0.981 | 0.007 | 0.863 | 0.053 |
| 7FCV | E+G+H+GE+GH | 2021 | Global | 0.989 | 0.001 | 0.941 | 0.005 |
| 7FCV | E+G+I | 2021 | Davenport | 0.950 | 0.007 | 0.898 | 0.017 |
| 7FCV | E+G+I | 2021 | Harrington | 0.974 | 0.011 | 0.955 | 0.020 |
| 7FCV | E+G+I | 2021 | Kahlotus | 0.942 | 0.030 | 0.930 | 0.034 |
| 7FCV | E+G+I | 2021 | Kincaid | 0.921 | 0.022 | 0.820 | 0.065 |
| 7FCV | E+G+I | 2021 | Lind | 0.956 | 0.007 | 0.938 | 0.008 |
| 7FCV | E+G+I | 2021 | Pullman | 0.000 | 0.000 | 0.015 | 0.073 |
| 7FCV | E+G+I | 2021 | Ritzville | 0.987 | 0.006 | 0.969 | 0.016 |
| 7FCV | E+G+I | 2021 | Walla_Walla | 0.949 | 0.010 | 0.869 | 0.025 |
| 7FCV | E+G+I | 2021 | Global | 0.968 | 0.003 | 0.962 | 0.003 |
| 7FCV | E+G+I+GE | 2021 | Davenport | 0.966 | 0.003 | 0.929 | 0.014 |
| 7FCV | E+G+I+GE | 2021 | Harrington | 0.980 | 0.011 | 0.964 | 0.011 |
| 7FCV | E+G+I+GE | 2021 | Kahlotus | 0.934 | 0.036 | 0.945 | 0.039 |
| 7FCV | E+G+I+GE | 2021 | Kincaid | 0.956 | 0.008 | 0.894 | 0.025 |
| 7FCV | E+G+I+GE | 2021 | Lind | 0.958 | 0.006 | 0.927 | 0.011 |
| 7FCV | E+G+I+GE | 2021 | Pullman | 0.000 | 0.000 | 0.109 | 0.067 |
| 7FCV | E+G+I+GE | 2021 | Ritzville | 0.989 | 0.005 | 0.963 | 0.017 |
| 7FCV | E+G+I+GE | 2021 | Walla_Walla | 0.963 | 0.006 | 0.918 | 0.021 |
| 7FCV | E+G+I+GE | 2021 | Global | 0.972 | 0.003 | 0.969 | 0.003 |
| 7FCV | E+G+I+GE+GI | 2021 | Davenport | 0.961 | 0.003 | 0.947 | 0.014 |
| 7FCV | E+G+I+GE+GI | 2021 | Harrington | 0.984 | 0.007 | 0.986 | 0.007 |
| 7FCV | E+G+I+GE+GI | 2021 | Kahlotus | 0.935 | 0.034 | 0.971 | 0.019 |
| 7FCV | E+G+I+GE+GI | 2021 | Kincaid | 0.952 | 0.009 | 0.854 | 0.035 |
| 7FCV | E+G+I+GE+GI | 2021 | Lind | 0.961 | 0.007 | 0.936 | 0.012 |
| 7FCV | E+G+I+GE+GI | 2021 | Pullman | 0.000 | 0.000 | 0.062 | 0.073 |
| 7FCV | E+G+I+GE+GI | 2021 | Ritzville | 0.990 | 0.004 | 0.695 | 0.283 |
| 7FCV | E+G+I+GE+GI | 2021 | Walla_Walla | 0.954 | 0.009 | 0.866 | 0.051 |
| 7FCV | E+G+I+GE+GI | 2021 | Global | 0.972 | 0.003 | 0.959 | 0.004 |
| 7FCV | E+H | 2021 | Davenport | 0.974 | 0.004 | 0.974 | 0.004 |
| 7FCV | E+H | 2021 | Harrington | 0.986 | 0.009 | 0.985 | 0.009 |
| 7FCV | E+H | 2021 | Kahlotus | 0.974 | 0.020 | 0.972 | 0.020 |
| 7FCV | E+H | 2021 | Kincaid | 0.968 | 0.006 | 0.968 | 0.006 |
| 7FCV | E+H | 2021 | Lind | 0.988 | 0.002 | 0.988 | 0.002 |
| 7FCV | E+H | 2021 | Pullman | 0.462 | 0.043 | 0.464 | 0.043 |
| 7FCV | E+H | 2021 | Ritzville | 0.990 | 0.003 | 0.689 | 0.282 |
| 7FCV | E+H | 2021 | Walla_Walla | 0.980 | 0.006 | 0.971 | 0.008 |
| 7FCV | E+H | 2021 | Global | 0.988 | 0.001 | 0.987 | 0.001 |
| 7FCV | E+I | 2021 | Davenport | 0.963 | 0.004 | 0.965 | 0.004 |
| 7FCV | E+I | 2021 | Harrington | 0.987 | 0.004 | 0.986 | 0.005 |
| 7FCV | E+I | 2021 | Kahlotus | 0.959 | 0.018 | 0.955 | 0.020 |
| 7FCV | E+I | 2021 | Kincaid | 0.948 | 0.006 | 0.957 | 0.006 |
| 7FCV | E+I | 2021 | Lind | 0.944 | 0.011 | 0.951 | 0.009 |
| 7FCV | E+I | 2021 | Pullman | 0.000 | 0.000 | 0.000 | 0.000 |
| 7FCV | E+I | 2021 | Ritzville | 0.994 | 0.003 | 0.991 | 0.004 |
| 7FCV | E+I | 2021 | Walla_Walla | 0.970 | 0.008 | 0.979 | 0.005 |
| 7FCV | E+I | 2021 | Global | 0.963 | 0.003 | 0.962 | 0.003 |

**Table A5.** Prediction performance for every environment and across environments (Global) of **dataset 4 (Year 2022)** in terms of Pearson´s correlation (Cor) for partially tested lines in tested environments (7FCV). Cor_GBLUP denotes the Cor computed under the GBLUP model, Cor_GBLUP_SE denotes its corresponding standard error. Cor_PLS denotes the Cor computed under the PLS model, Cor_PLS_SE denotes its corresponding standard error.

| CV | Predictor | Year | Env | Cor_GBLUP | Cor_GBLUP_SE | Cor_PLS | Cor_PLS_SE |
| --- | --- | --- | --- | --- | --- | --- | --- |
| 7FCV | E+G | 2022 | Farmington | 0.139 | 0.266 | 0.470 | 0.150 |
| 7FCV | E+G | 2022 | Harrington | 0.368 | 0.297 | 0.157 | 0.250 |
| 7FCV | E+G | 2022 | Prescott | 0.704 | 0.160 | 0.000 | 0.000 |
| 7FCV | E+G | 2022 | Pullman | 0.517 | 0.058 | 0.344 | 0.094 |
| 7FCV | E+G | 2022 | Global | 0.826 | 0.021 | 0.792 | 0.029 |
| 7FCV | E+G+H | 2022 | Farmington | 0.000 | 0.000 | 0.248 | 0.350 |
| 7FCV | E+G+H | 2022 | Harrington | 0.645 | 0.175 | 0.160 | 0.354 |
| 7FCV | E+G+H | 2022 | Prescott | 0.895 | 0.080 | 0.000 | 0.000 |
| 7FCV | E+G+H | 2022 | Pullman | 0.529 | 0.053 | 0.482 | 0.048 |
| 7FCV | E+G+H | 2022 | Global | 0.833 | 0.019 | 0.813 | 0.018 |
| 7FCV | E+G+H+GE | 2022 | Farmington | 0.000 | 0.000 | 0.258 | 0.350 |
| 7FCV | E+G+H+GE | 2022 | Harrington | 0.701 | 0.155 | 0.505 | 0.293 |
| 7FCV | E+G+H+GE | 2022 | Prescott | 0.901 | 0.067 | 0.000 | 0.000 |
| 7FCV | E+G+H+GE | 2022 | Pullman | 0.518 | 0.054 | 0.490 | 0.047 |
| 7FCV | E+G+H+GE | 2022 | Global | 0.831 | 0.018 | 0.816 | 0.018 |
| 7FCV | E+G+H+GE+GH | 2022 | Farmington | 0.000 | 0.000 | 0.490 | 0.206 |
| 7FCV | E+G+H+GE+GH | 2022 | Harrington | 0.006 | 0.274 | 0.216 | 0.318 |
| 7FCV | E+G+H+GE+GH | 2022 | Prescott | 0.896 | 0.073 | 0.629 | 0.185 |
| 7FCV | E+G+H+GE+GH | 2022 | Pullman | 0.518 | 0.055 | 0.212 | 0.065 |
| 7FCV | E+G+H+GE+GH | 2022 | Global | 0.829 | 0.019 | 0.577 | 0.043 |
| 7FCV | E+G+I | 2022 | Farmington | 0.000 | 0.000 | 0.507 | 0.156 |
| 7FCV | E+G+I | 2022 | Harrington | 0.331 | 0.294 | 0.496 | 0.258 |
| 7FCV | E+G+I | 2022 | Prescott | 0.902 | 0.069 | 0.030 | 0.339 |
| 7FCV | E+G+I | 2022 | Pullman | 0.528 | 0.055 | 0.374 | 0.091 |
| 7FCV | E+G+I | 2022 | Global | 0.832 | 0.019 | 0.794 | 0.029 |
| 7FCV | E+G+I+GE | 2022 | Farmington | 0.000 | 0.000 | 0.525 | 0.168 |
| 7FCV | E+G+I+GE | 2022 | Harrington | 0.387 | 0.282 | 0.542 | 0.263 |
| 7FCV | E+G+I+GE | 2022 | Prescott | 0.915 | 0.051 | 0.379 | 0.302 |
| 7FCV | E+G+I+GE | 2022 | Pullman | 0.518 | 0.055 | 0.355 | 0.087 |
| 7FCV | E+G+I+GE | 2022 | Global | 0.831 | 0.019 | 0.789 | 0.030 |
| 7FCV | E+G+I+GE+GI | 2022 | Farmington | 0.000 | 0.000 | 0.441 | 0.234 |
| 7FCV | E+G+I+GE+GI | 2022 | Harrington | 0.000 | 0.000 | 0.572 | 0.203 |
| 7FCV | E+G+I+GE+GI | 2022 | Prescott | 0.914 | 0.053 | 0.612 | 0.213 |
| 7FCV | E+G+I+GE+GI | 2022 | Pullman | 0.516 | 0.057 | 0.451 | 0.055 |
| 7FCV | E+G+I+GE+GI | 2022 | Global | 0.829 | 0.019 | 0.769 | 0.017 |
| 7FCV | E+H | 2022 | Farmington | 0.083 | 0.352 | 0.073 | 0.369 |
| 7FCV | E+H | 2022 | Harrington | 0.369 | 0.291 | 0.000 | 0.000 |
| 7FCV | E+H | 2022 | Prescott | 0.574 | 0.259 | 0.000 | 0.000 |
| 7FCV | E+H | 2022 | Pullman | 0.359 | 0.062 | 0.349 | 0.044 |
| 7FCV | E+H | 2022 | Global | 0.796 | 0.016 | 0.788 | 0.010 |
| 7FCV | E+I | 2022 | Farmington | 0.103 | 0.347 | 0.000 | 0.000 |
| 7FCV | E+I | 2022 | Harrington | 0.028 | 0.338 | 0.012 | 0.330 |
| 7FCV | E+I | 2022 | Prescott | 0.304 | 0.317 | 0.564 | 0.294 |
| 7FCV | E+I | 2022 | Pullman | 0.360 | 0.064 | 0.257 | 0.058 |
| 7FCV | E+I | 2022 | Global | 0.796 | 0.016 | 0.772 | 0.015 |

**Table A6.** Prediction performance for every environment and across environments (Global) of **dataset 1 (Year 2019)** in terms of Pearson´s correlation (Cor) for partially tested lines in untested environments (LOEO). Cor_GBLUP denotes the Cor computed under the GBLUP model, Cor_GBLUP_SE denotes its corresponding standard error. Cor_PLS denotes the Cor computed under the PLS model, Cor_PLS_SE denotes its corresponding standard error.

| CV | Predictor | Year | Env | Cor_GBLUP | Cor_GBLUP_SE | Cor_PLS | Cor_PLS_SE |
| --- | --- | --- | --- | --- | --- | --- | --- |
| LOEO | G | 2019 | Kincaid | 0.109 | NA | 0.000 | 0.000 |
| LOEO | G | 2019 | Lind | 0.000 | 0.000 | 0.000 | 0.000 |
| LOEO | G | 2019 | Pullman | 0.029 | NA | 0.004 | NA |
| LOEO | G | 2019 | Global | 0.000 | 0.000 | 0.000 | 0.000 |
| LOEO | G+H | 2019 | Kincaid | 0.755 | NA | 0.921 | NA |
| LOEO | G+H | 2019 | Lind | 0.520 | NA | 0.531 | NA |
| LOEO | G+H | 2019 | Pullman | 0.489 | NA | 0.313 | NA |
| LOEO | G+H | 2019 | Global | 0.588 | 0.084 | 0.588 | 0.178 |
| LOEO | G+I | 2019 | Kincaid | 0.958 | NA | 0.957 | NA |
| LOEO | G+I | 2019 | Lind | 0.381 | NA | 0.564 | NA |
| LOEO | G+I | 2019 | Pullman | 0.570 | NA | 0.510 | NA |
| LOEO | G+I | 2019 | Global | 0.637 | 0.170 | 0.677 | 0.141 |
| LOEO | H | 2019 | Kincaid | 0.798 | NA | 0.959 | NA |
| LOEO | H | 2019 | Lind | 0.641 | NA | 0.617 | NA |
| LOEO | H | 2019 | Pullman | 0.331 | NA | 0.433 | NA |
| LOEO | H | 2019 | Global | 0.590 | 0.137 | 0.670 | 0.154 |
| LOEO | I | 2019 | Kincaid | 0.959 | NA | 0.959 | NA |
| LOEO | I | 2019 | Lind | 0.621 | NA | 0.617 | NA |
| LOEO | I | 2019 | Pullman | 0.535 | NA | 0.434 | NA |
| LOEO | I | 2019 | Global | 0.705 | 0.129 | 0.670 | 0.154 |

**Table A7.** Prediction performance for every environment and across environments (Global) of **dataset 2 (Year 2020)** in terms of Pearson´s correlation (Cor) for partially tested lines in untested environments (LOEO). Cor_GBLUP denotes the Cor computed under the GBLUP model, Cor_GBLUP_SE denotes its corresponding standard error. Cor_PLS denotes the Cor computed under the PLS model, Cor_PLS_SE denotes its corresponding standard error.

| CV | Predictor | Year | Env | Cor_GBLUP | Cor_GBLUP_SE | Cor_PLS | Cor_PLS_SE |
| --- | --- | --- | --- | --- | --- | --- | --- |
| LOEO | G | 2020 | Farmington | 0.035 | NA | 0.000 | 0.000 |
| LOEO | G | 2020 | Harrington | 0.000 | 0.000 | 0.000 | 0.000 |
| LOEO | G | 2020 | Kincaid | 0.000 | 0.000 | 0.000 | 0.000 |
| LOEO | G | 2020 | Lind | 0.000 | 0.000 | 0.000 | 0.000 |
| LOEO | G | 2020 | Ritzville | 0.110 | NA | 0.279 | NA |
| LOEO | G | 2020 | Walla_Walla | 0.000 | 0.000 | 0.000 | 0.000 |
| LOEO | G | 2020 | Global | 0.000 | 0.000 | 0.000 | 0.000 |
| LOEO | G+H | 2020 | Farmington | 0.905 | NA | 0.854 | NA |
| LOEO | G+H | 2020 | Harrington | 0.967 | NA | 0.733 | NA |
| LOEO | G+H | 2020 | Kincaid | 0.152 | NA | 0.169 | NA |
| LOEO | G+H | 2020 | Lind | 0.092 | NA | 0.504 | NA |
| LOEO | G+H | 2020 | Ritzville | 0.897 | NA | 0.825 | NA |
| LOEO | G+H | 2020 | Walla_Walla | 0.895 | NA | 0.924 | NA |
| LOEO | G+H | 2020 | Global | 0.651 | 0.168 | 0.668 | 0.116 |
| LOEO | G+I | 2020 | Farmington | 0.848 | NA | 0.758 | NA |
| LOEO | G+I | 2020 | Harrington | 0.938 | NA | 0.928 | NA |
| LOEO | G+I | 2020 | Kincaid | 0.133 | NA | 0.190 | NA |
| LOEO | G+I | 2020 | Lind | 0.265 | NA | 0.572 | NA |
| LOEO | G+I | 2020 | Ritzville | 0.904 | NA | 0.921 | NA |
| LOEO | G+I | 2020 | Walla_Walla | 0.828 | NA | 0.697 | NA |
| LOEO | G+I | 2020 | Global | 0.653 | 0.145 | 0.678 | 0.112 |
| LOEO | H | 2020 | Farmington | 0.934 | NA | 0.881 | NA |
| LOEO | H | 2020 | Harrington | 0.974 | NA | 0.967 | NA |
| LOEO | H | 2020 | Kincaid | 0.165 | NA | 0.195 | NA |
| LOEO | H | 2020 | Lind | 0.098 | NA | 0.725 | NA |
| LOEO | H | 2020 | Ritzville | 0.920 | NA | 0.938 | NA |
| LOEO | H | 2020 | Walla_Walla | 0.951 | NA | 0.928 | NA |
| LOEO | H | 2020 | Global | 0.674 | 0.172 | 0.772 | 0.121 |
| LOEO | I | 2020 | Farmington | 0.896 | NA | 0.897 | NA |
| LOEO | I | 2020 | Harrington | 0.954 | NA | 0.953 | NA |
| LOEO | I | 2020 | Kincaid | 0.146 | NA | 0.146 | NA |
| LOEO | I | 2020 | Lind | 0.291 | NA | 0.274 | NA |
| LOEO | I | 2020 | Ritzville | 0.945 | NA | 0.946 | NA |
| LOEO | I | 2020 | Walla_Walla | 0.902 | NA | 0.902 | NA |
| LOEO | I | 2020 | Global | 0.689 | 0.150 | 0.686 | 0.152 |

**Table A8.** Prediction performance for every environment and across environments (Global) of **dataset 3 (Year 2021)** in terms of Pearson´s correlation (Cor) for partially tested lines in untested environments (LOEO). Cor_GBLUP denotes the Cor computed under the GBLUP model, Cor_GBLUP_SE denotes its corresponding standard error. Cor_PLS denotes the Cor computed under the PLS model, Cor_PLS_SE denotes its corresponding standard error.

| CV | Predictor | Year | Env | Cor_GBLUP | Cor_GBLUP_SE | Cor_PLS | Cor_PLS_SE |
| --- | --- | --- | --- | --- | --- | --- | --- |
| LOEO | G | 2021 | Davenport | 0.085 | NA | 0.048 | NA |
| LOEO | G | 2021 | Harrington | 0.779 | NA | 0.625 | NA |
| LOEO | G | 2021 | Kahlotus | 0.595 | NA | 0.465 | NA |
| LOEO | G | 2021 | Kincaid | 0.410 | NA | 0.397 | NA |
| LOEO | G | 2021 | Lind | 0.530 | NA | 0.248 | NA |
| LOEO | G | 2021 | Pullman | 0.000 | 0.000 | 0.000 | 0.000 |
| LOEO | G | 2021 | Ritzville | 0.683 | NA | 0.612 | NA |
| LOEO | G | 2021 | Walla_Walla | 0.509 | NA | 0.380 | NA |
| LOEO | G | 2021 | Global | 0.435 | 0.107 | 0.331 | 0.093 |
| LOEO | G+H | 2021 | Davenport | 0.931 | NA | 0.909 | NA |
| LOEO | G+H | 2021 | Harrington | 0.952 | NA | 0.933 | NA |
| LOEO | G+H | 2021 | Kahlotus | 0.931 | NA | 0.934 | NA |
| LOEO | G+H | 2021 | Kincaid | 0.952 | NA | 0.955 | NA |
| LOEO | G+H | 2021 | Lind | 0.966 | NA | 0.966 | NA |
| LOEO | G+H | 2021 | Pullman | 0.000 | 0.000 | 0.000 | 0.000 |
| LOEO | G+H | 2021 | Ritzville | 0.954 | NA | 0.957 | NA |
| LOEO | G+H | 2021 | Walla_Walla | 0.967 | NA | 0.976 | NA |
| LOEO | G+H | 2021 | Global | 0.786 | 0.165 | 0.793 | 0.155 |
| LOEO | G+I | 2021 | Davenport | 0.935 | NA | 0.907 | NA |
| LOEO | G+I | 2021 | Harrington | 0.976 | NA | 0.938 | NA |
| LOEO | G+I | 2021 | Kahlotus | 0.957 | NA | 0.880 | NA |
| LOEO | G+I | 2021 | Kincaid | 0.836 | NA | 0.766 | NA |
| LOEO | G+I | 2021 | Lind | 0.874 | NA | 0.755 | NA |
| LOEO | G+I | 2021 | Pullman | 0.000 | 0.000 | 0.000 | 0.000 |
| LOEO | G+I | 2021 | Ritzville | 0.967 | NA | 0.917 | NA |
| LOEO | G+I | 2021 | Walla_Walla | 0.910 | NA | 0.844 | NA |
| LOEO | G+I | 2021 | Global | 0.755 | 0.168 | 0.700 | 0.160 |
| LOEO | H | 2021 | Davenport | 0.927 | NA | 0.914 | NA |
| LOEO | H | 2021 | Harrington | 0.952 | NA | 0.952 | NA |
| LOEO | H | 2021 | Kahlotus | 0.946 | NA | 0.943 | NA |
| LOEO | H | 2021 | Kincaid | 0.960 | NA | 0.960 | NA |
| LOEO | H | 2021 | Lind | 0.970 | NA | 0.966 | NA |
| LOEO | H | 2021 | Pullman | 0.000 | 0.000 | 0.000 | 0.000 |
| LOEO | H | 2021 | Ritzville | 0.969 | NA | 0.972 | NA |
| LOEO | H | 2021 | Walla_Walla | 0.979 | NA | 0.975 | NA |
| LOEO | H | 2021 | Global | 0.786 | 0.172 | 0.785 | 0.170 |
| LOEO | I | 2021 | Davenport | 0.967 | NA | 0.969 | NA |
| LOEO | I | 2021 | Harrington | 0.985 | NA | 0.986 | NA |
| LOEO | I | 2021 | Kahlotus | 0.971 | NA | 0.973 | NA |
| LOEO | I | 2021 | Kincaid | 0.966 | NA | 0.970 | NA |
| LOEO | I | 2021 | Lind | 0.852 | NA | 0.724 | NA |
| LOEO | I | 2021 | Pullman | 0.000 | 0.000 | 0.000 | 0.000 |
| LOEO | I | 2021 | Ritzville | 0.987 | NA | 0.989 | NA |
| LOEO | I | 2021 | Walla_Walla | 0.974 | NA | 0.983 | NA |
| LOEO | I | 2021 | Global | 0.786 | 0.172 | 0.772 | 0.173 |

**Table A9.** Prediction performance for every environment and across environments (Global) of **dataset 4 (Year 2022)** in terms of Pearson´s correlation (Cor) for partially tested lines in untested environments (LOEO). Cor_GBLUP denotes the Cor computed under the GBLUP model, Cor_GBLUP_SE denotes its corresponding standard error. Cor_PLS denotes the Cor computed under the PLS model, Cor_PLS_SE denotes its corresponding standard error.

| CV | Predictor | Year | Env | Cor_GBLUP | Cor_GBLUP_SE | Cor_PLS | Cor_PLS_SE |
| --- | --- | --- | --- | --- | --- | --- | --- |
| LOEO | G | 2022 | Farmington | 0.166 | NA | 0.058 | NA |
| LOEO | G | 2022 | Harrington | 0.365 | NA | 0.248 | NA |
| LOEO | G | 2022 | Prescott | 0.293 | NA | 0.111 | NA |
| LOEO | G | 2022 | Pullman | 0.130 | NA | 0.064 | NA |
| LOEO | G | 2022 | Global | 0.239 | 0.055 | 0.120 | 0.044 |
| LOEO | G+H | 2022 | Farmington | 0.117 | NA | 0.420 | NA |
| LOEO | G+H | 2022 | Harrington | 0.538 | NA | 0.000 | 0.000 |
| LOEO | G+H | 2022 | Prescott | 0.581 | NA | 0.323 | NA |
| LOEO | G+H | 2022 | Pullman | 0.355 | NA | 0.288 | NA |
| LOEO | G+H | 2022 | Global | 0.398 | 0.106 | 0.189 | 0.157 |
| LOEO | G+I | 2022 | Farmington | 0.216 | NA | 0.295 | NA |
| LOEO | G+I | 2022 | Harrington | 0.322 | NA | 0.185 | NA |
| LOEO | G+I | 2022 | Prescott | 0.475 | NA | 0.128 | NA |
| LOEO | G+I | 2022 | Pullman | 0.389 | NA | 0.067 | NA |
| LOEO | G+I | 2022 | Global | 0.351 | 0.055 | 0.169 | 0.049 |
| LOEO | H | 2022 | Farmington | 0.145 | NA | 0.427 | NA |
| LOEO | H | 2022 | Harrington | 0.053 | NA | 0.000 | 0.000 |
| LOEO | H | 2022 | Prescott | 0.274 | NA | 0.154 | NA |
| LOEO | H | 2022 | Pullman | 0.306 | NA | 0.288 | NA |
| LOEO | H | 2022 | Global | 0.195 | 0.059 | 0.112 | 0.187 |
| LOEO | I | 2022 | Farmington | 0.556 | NA | 0.448 | NA |
| LOEO | I | 2022 | Harrington | 0.500 | NA | 0.479 | NA |
| LOEO | I | 2022 | Prescott | 0.196 | NA | 0.190 | NA |
| LOEO | I | 2022 | Pullman | 0.318 | NA | 0.335 | NA |
| LOEO | I | 2022 | Global | 0.393 | 0.083 | 0.363 | 0.066 |
